# Development and internal validation of CTA-based radiomics models for differentiating symptomatic from asymptomatic unruptured spontaneous intracranial vertebrobasilar artery dissection

**DOI:** 10.3389/fneur.2026.1883223

**Published:** 2026-10-23

**Authors:** Jia Jiang, Sheng Bi, Siming Gui, Lu Zheng, Youxiang Li, Qinglin Liu

**Affiliations:** Department of Neurosurgery, Beijing Neurosurgical Institute and Beijing Tiantan Hospital, Capital Medical University, Beijing, China

**Keywords:** computed tomography angiography, machine learning, radiomics, symptomatic status, vertebrobasilar artery dissection

## Abstract

**Background:**

Spontaneous intracranial vertebrobasilar artery dissection (sIVBD) has heterogeneous clinical presentations, and conventional clinical and morphologic assessment may not fully characterize lesion heterogeneity. We aimed to develop and internally validate CTA-based radiomics models for differentiating symptomatic from asymptomatic unruptured sIVBD and to evaluate the incremental contribution of radiomics beyond conventional clinical and CTA features.

**Methods:**

This retrospective single-center study included 173 patients with unruptured sIVBD, comprising 56 symptomatic and 117 asymptomatic patients. Patients were stratified into a development cohort (*n* = 138) and an independent internal validation cohort (*n* = 35). Radiomic features were extracted from subtraction CTA images after three-dimensional lesion segmentation. Feature reproducibility was assessed using inter-reader intraclass correlation coefficients. Four models were developed: a conventional CTA model (M1), a clinical plus conventional CTA model (M2), a radiomics XGBoost model (M3), and a combined model integrating M2 predictors with a cross-fitted radiomics probability (M4). Primary development-cohort evaluation used five-times-repeated five-fold stratified nested cross-validation. Discrimination, calibration, Brier score, and decision-curve analysis were assessed.

**Results:**

Of 851 radiomic features, 722 (84.8%) met the prespecified reproducibility criterion. In patient-averaged out-of-fold evaluation, AUCs were 0.630 for M1, 0.681 for M2, 0.676 for M3, and 0.723 for M4. Compared with M2, M4 increased AUC by 0.0416 (95% CI, −0.0130 to 0.0962; *p* = 0.136) and reduced the Brier score by 0.0101 (95% bootstrap CI, −0.0208 to 0.0006). Across 25 outer folds, M4 had the highest mean AUC (0.715) and the lowest between-fold variability. In the independent internal validation cohort, AUCs were 0.814, 0.807, 0.803, and 0.966 for M1 through M4, respectively.

**Conclusion:**

A combined model integrating clinical variables, conventional CTA features, and cross-fitted radiomics information showed the best overall performance for differentiating symptomatic from asymptomatic unruptured sIVBD. Radiomics may provide complementary quantitative information beyond conventional assessment.

## Introduction

Spontaneous intracranial vertebrobasilar artery dissection (sIVBD) is characterized by disruption of the arterial wall with intramural hematoma formation, resulting in a spectrum of luminal and vascular wall abnormalities, including stenosis, aneurysmal dilatation, and other morphological changes (1–3). Its clinical presentation is highly variable, ranging from incidental or minimally symptomatic disease to posterior circulation ischemic stroke, subarachnoid hemorrhage (SAH), and symptoms related to brainstem compression. This heterogeneity makes sIVBD an important and clinically challenging cerebrovascular disorder, particularly in young and middle-aged adults (2, 4).

The natural history of sIVBD is similarly heterogeneous and remains difficult to predict. Some lesions may remain stable or improve spontaneously, whereas others may be associated with ischemic events due to hemodynamic compromise or thromboembolism, rupture with subarachnoid hemorrhage, or progressive enlargement with mass effect and neurological deterioration. Although several clinical and imaging characteristics, including smoking history, intraluminal thrombus, prior subarachnoid hemorrhage, and lesion size or volume, have been associated with adverse clinical or imaging features, their ability to support individualized clinical assessment remains limited (2).

This uncertainty has direct implications for management. Treatment decisions require integration of symptoms, lesion morphology, vascular anatomy, and the potential risks of intervention, particularly because endovascular treatment of vertebrobasilar dissections may itself carry substantial procedural risk (5, 6). Conventional clinical and imaging assessment therefore remains essential but may not fully capture subtle lesion heterogeneity. More objective and quantitative characterization of lesion morphology and texture could provide complementary information beyond routinely assessed imaging features (7).

Radiomics enables high-throughput extraction of quantitative imaging features that may capture lesion characteristics not readily appreciated by visual assessment and has increasingly been applied to intracranial vascular lesions (8, 9). However, the utility of Computed Tomography Angiography (CTA)-based radiomics for differentiating symptomatic from asymptomatic unruptured sIVBD remains insufficiently investigated. Therefore, this study aimed to develop and internally validate CTA-based radiomics models for differentiating symptomatic from asymptomatic unruptured sIVBD and to evaluate their discrimination, calibration, clinical utility, and interpretability.

## Materials and methods

### Study design and participants

This single-center retrospective study was reviewed and approved by the Institutional Review Board of our institution and was conducted in accordance with the Declaration of Helsinki. The requirement for written informed consent was waived by the Institutional Review Board owing to the retrospective nature of the study. The study was reported in accordance with the STROBE statement. Consecutive patients with sIVBD who were evaluated between May 2018 and September 2024 were screened for eligibility. The final cohort included 173 patients with sIVBD, comprising 56 symptomatic and 117 asymptomatic patients.

The inclusion criteria were as follows: (1) age between 18 and 75 years; (2) diagnosis of unruptured sIVBD based on CTA, digital subtraction angiography (DSA), or magnetic resonance imaging (MRI); (3) availability of pretreatment CTA images of sufficient quality for lesion segmentation and radiomic feature extraction; and (4) sufficient clinical information to determine symptomatic status. The exclusion criteria were as follows: (1) ruptured sIVBD or SAH attributable to the target lesion at presentation; (2) traumatic, infectious, or iatrogenic arterial dissection; (3) coexisting intracranial tumors, cerebrovascular malformations, or other conditions substantially affecting the target vascular lesion; (4) prior treatment of the target lesion before the CTA examination used for radiomics analysis; and (5) incomplete clinical data or CTA images unsuitable for radiomic analysis. Patient inclusion was based on the predefined eligibility criteria and was independent of the subsequent treatment strategy.

### Demographic clinical and morphological data collection

Demographic and clinical characteristics included age, sex, comorbidities (history of hypertension, dyslipidemia, diabetes mellitus, coronary artery disease, cerebral infarction, and non-sIVBD-related SAH), smoking status, and alcohol consumption. Conventional morphological characteristics included lesion location(Basilar artery(BA) involvement), length, maximum width, and the presence of parent artery stenosis. Age was unavailable for 18 of 173 patients (10.4%) and was replaced with the median age of the overall cohort (57 years) during preparation of the clinical dataset. Height and weight also contained substantial missing data and were excluded before model specification; neither the original nor imputed height and weight values were used in any of the four models. The conventional CTA variables used for modeling, including lesion location, lesion length, maximum width, and parent artery stenosis, were complete. Radiomic feature extraction was completed for all included lesions, with no missing values among the extracted radiomic features.

### Diagnosis of sIVBD and sIVBD-related symptoms

Diagnostic criteria of sIVBD included one or more of the following findings: (1) segmental irregularity, stenosis, or fusiform/aneurysmal dilatation of the intracranial vertebral or basilar artery; (2) a pearl-and-string or string sign; (3) contrast retention within the dissected segment on DSA; or (4) an intramural hematoma, intimal flap, or double-lumen sign on MRI (10, 11). The primary endpoint of this study was symptomatic status at the time of clinical evaluation, representing a cross-sectional classification endpoint rather than longitudinal lesion stability or future progression. Patients were classified as symptomatic based on clinical manifestations attributable to the sIVBD lesion, including ischemic neurological events, dissection-related pain/headache, or symptoms related to local mass effect. Radiological enlargement was not used as an independent criterion for symptomatic classification and was recorded separately as a longitudinal imaging finding when serial vascular imaging was available, based on comparison with prior vascular imaging examinations rather than the radiomics CTA examination alone.

### CTA acquisition and reconstruction

All CTA examinations were performed using a Revolution CT system (GE Healthcare, Milwaukee, WI, USA) with a standardized subtraction CTA protocol. The scan range extended from the C2 level to the vertex. Axial acquisitions were obtained at a tube voltage of 80 kVp and a tube current of 150 mA. The gantry rotation time was 1.0 s, with a detector collimation of 256 × 0.625 mm. Images were reconstructed with a section thickness of 0.625 mm using a standard reconstruction kernel and medium filter. The reconstruction field of view was 250 mm with a 512 × 512 matrix, corresponding to an in-plane pixel spacing of approximately 0.49 × 0.49 mm.

For contrast-enhanced CTA, a nonionic iodinated contrast medium with an iodine concentration of 350–370 mg I/mL was administered intravenously. A test-bolus technique was used to determine the individual time to peak vascular enhancement. Specifically, 15 mL of contrast medium was injected at a rate of 6.0 mL/s for the test bolus. The subsequent CTA acquisition was performed using 55 mL of contrast medium injected at 6.0 mL/s, with the scan delay individualized according to the time to peak enhancement determined from the test bolus.

The subtraction CTA protocol consisted of sequential noncontrast and contrast-enhanced acquisitions. The noncontrast dataset was deformably registered to the contrast-enhanced dataset to compensate for interscan motion, after which subtraction images were generated by removing the registered noncontrast signal from the contrast-enhanced images. The resulting thin-section axial subtraction CTA dataset was exported in Digital Imaging and Communications in Medicine (DICOM) format and used for subsequent three-dimensional vascular reconstruction, lesion segmentation, and radiomics analysis.

### Lesion segmentation

The axial subtraction CTA datasets in DICOM format were imported into 3D Slicer (version 5.6.2) for lesion segmentation. Initial vascular segmentation was performed using a threshold-based approach, followed by three-dimensional vascular reconstruction. Vessels and structures outside the target dissecting lesion were manually removed and refined with reference to the axial and multiplanar images, and the resulting three-dimensional segmentation of the entire sIVBD lesion was retained as the region of interest (ROI).

Three experienced neuroimaging specialists independently segmented all lesions using the same predefined segmentation protocol. The ROI generated by the senior reader (Reader 1, with more than 10 years of experience in neurovascular imaging) was used for the primary radiomic feature extraction and subsequent model development. Segmentations generated independently by Readers 2 and 3 were retained for assessment of inter-reader reproducibility(ICC) (12).

Feature-level inter-reader reproducibility was assessed exclusively in the development cohort using ICC(2,1), corresponding to a two-way random-effects, absolute-agreement, single-measure model based on the three reader-specific feature measurements. Features with an ICC point estimate ≥0.75 were retained. Because this reproducibility assessment did not use symptomatic-status labels, the ICC filter was determined once using the full development cohort before repeated nested cross-validation and was not recalculated within individual outer folds. Feature values derived from the Reader 1 segmentation were used for all subsequent model development.

### Radiomics preprocessing and feature extraction

Radiomic feature extraction was performed using PyRadiomics implemented in 3D Slicer (version 5.6.2) through the SlicerRadiomics module. Manual customization was used for feature-extraction settings. Before feature extraction, the subtraction CTA images and corresponding segmentation masks were resampled to an isotropic voxel spacing of 1 × 1 × 1 mm^3^. B-spline interpolation was used for image resampling, whereas nearest-neighbor interpolation was used for segmentation masks. No image-intensity normalization was applied. Gray levels were discretized using a fixed bin width of 25.

Radiomic features were extracted from the original subtraction CTA images and eight wavelet-transformed images. The extracted feature classes included 14 shape features, 18 first-order features, 24 gray-level co-occurrence matrix (GLCM) features, 16 gray-level run-length matrix (GLRLM) features, 16 gray-level size-zone matrix (GLSZM) features, 14 gray-level dependence matrix (GLDM) features, and 5 neighboring gray-tone difference matrix (NGTDM) features. Symmetrical GLCM calculation was enabled. Laplacian-of-Gaussian filtering was not applied.

First-order and texture features were calculated from the original image and eight wavelet-decomposed images, whereas shape features were calculated from the original lesion segmentation. In total, 851 radiomic features were extracted for each lesion, comprising 14 shape features and 837 first-order and texture features derived from the original and wavelet-transformed images.

Features passing the ICC filter underwent quality control, Mann–Whitney U screening, Spearman correlation filtering, mRMR selection, and LASSO logistic regression within the corresponding training partitions. Univariate screening used a two-sided *p* value <0.05, and redundancy filtering used an absolute Spearman correlation threshold of 0.90. mRMR employed the mutual information difference criterion to retain up to 50 features. LASSO selected nonzero-coefficient features using the λ minimizing mean validation binomial deviance in five-fold stratified cross-validation. Imputation and standardization were fitted within the corresponding LASSO training folds. Outcome-informed screening and selection were repeated within the training subsets used for radiomics development and hyperparameter evaluation; the feature set selected from the entire development cohort was not fixed across outer validation partitions. Detailed processing rules are provided in Supplementary methods S1.

### Model development and hyperparameter optimization

The 173 patients were stratified by symptomatic status and randomly allocated at an approximately 8:2 ratio to a development cohort of 138 patients and an independent internal validation cohort of 35 patients. All model-development procedures, including radiomic reproducibility assessment, feature selection, preprocessing, hyperparameter optimization, and threshold determination, were conducted using the development cohort. The independent internal validation cohort was evaluated only after the final models and thresholds had been locked.

Four models were developed using prespecified predictors. M1 included lesion location, length, maximum width, and parent artery stenosis. M2 added age, sex, smoking, alcohol consumption, hypertension, diabetes mellitus, coronary artery disease, and dyslipidemia. M3 was an XGBoost radiomics model. M4 combined the radiomics-derived probability with the 12 predictors in M2. Clinical and conventional CTA predictors were not selected using univariable group-comparison *p* values. M1, M2, and M4 used ridge logistic regression. Continuous predictors were standardized using parameters estimated from the corresponding training partition, whereas binary predictors retained their 0/1 coding. After the clinical-data preparation described above, no unresolved missing values remained among the predictors entered into M1, M2, and M4. Median imputation for continuous variables and mode imputation for categorical variables remained prespecified within the modeling pipeline as safeguards for potential missing values, but no additional imputation was triggered for the finalized model-input dataset. The regularization parameter C was selected by minimizing five-fold internal-validation log-loss. M3 hyperparameters were optimized using GWO (13), with 25 wolves and 60 fixed iterations. Candidate fitness was the mean ROC-AUC across five inner validation folds. Grid Search was evaluated using identical outer and inner partitions and the same inner-training feature-selection results. GWO and Grid Search evaluated 1,500 and 8,748 candidate configurations per outer partition, respectively. Their search spaces and budgets differed; the comparison was not designed to establish superiority under equal computational resources. Complete optimization settings are provided in Supplementary methods S2. M4 used five-fold cross-fitted radiomics probabilities: each training patient’s score was generated by an upstream model developed without that patient’s outcome. During internal selection of the M4 penalty, training scores were cross-fitted within the internal training subset, whereas validation scores were generated by an upstream model fitted exclusively on that training subset. After penalty selection, fresh cross-fitted probabilities were generated for the complete current training partition to fit M4. For held-out patients, probabilities from the upstream model fitted on the complete corresponding training partition were entered into M4. This procedure is detailed in Supplementary methods S3. A nomogram visualized the final combined model.

### Model validation and interpretation

Primary evaluation used five-times-repeated five-fold stratified nested cross-validation in the development cohort. Each patient received five out-of-fold predictions per model, which were averaged at the patient level to calculate primary performance. Repeat-specific and outer-fold performance were additionally summarized. Final models were fitted on all 138 development patients and subsequently evaluated in the independent internal validation cohort.

Performance measures included ROC-AUC with 95% confidence intervals, average precision, Brier score, calibration-in-the-large, calibration slope, and calibration intercept. To quantify uncertainty in model performance estimates, 95% confidence intervals were additionally reported for calibration-in-the-large, calibration slope, and jointly estimated calibration intercept using profile-likelihood methods. Calibration metrics were evaluated based on the original model predictions without any updating or recalibration in the independent internal validation cohort. Calibration curves were generated for evaluation and visualization of agreement between predicted probabilities and observed outcomes. Model-specific classification thresholds maximized the Youden index in patient-averaged development out-of-fold predictions and were applied unchanged to the independent internal validation cohort. Confusion-matrix counts and classification measures were reported with Wilson 95% confidence intervals.

Decision curve analysis assessed exploratory net benefit relative to treat-all and treat-none strategies. SHAP analysis interpreted the final M3 model (14), and feature-selection frequencies and pairwise Jaccard overlap across outer partitions described selection stability. Computational details are provided in Supplementary methods S4.

### Statistical analysis

Continuous variables were summarized as mean ± standard deviation or median with interquartile range, according to their distribution, and compared using two-sided Mann–Whitney U tests. Categorical variables were reported as counts and percentages and compared using Pearson’s chi-square or Fisher’s exact test, as appropriate. Standardized mean differences described differences between the development and independent internal validation cohorts.

The prespecified primary comparison was M4 versus M2. Paired DeLong analyses compared patient-averaged out-of-fold AUCs. Secondary comparisons were M2 versus M1, M3 versus M1, and M4 versus M3, with Holm adjustment across these three tests. GWO-XGBoost and Grid-XGBoost were compared separately using the same paired approach.

The M4-minus-M2 Brier difference was evaluated using 2,000 stratified paired patient-level bootstrap samples, with percentile confidence intervals. This procedure resampled fixed predictions without repeating model development. Statistical comparisons were therefore interpreted conditionally on the generated predictions. Tests were two-sided, with *p* < 0.05 considered statistically significant, subject to multiplicity adjustment. Analyses used Python(3.13.5), scikit-learn 1.8.0, and XGBoost 3.1.2; additional implementation details and software versions are provided in Supplementary methods S2, S4.

## Results

### Study population and baseline characteristics

A total of 173 patients were included, comprising 117 asymptomatic and 56 symptomatic patients. After stratified random partitioning, 138 patients were assigned to the development cohort, including 93 asymptomatic and 45 symptomatic patients, and 35 patients were assigned to the independent internal validation cohort, including 24 asymptomatic and 11 symptomatic patients (Figure 1). Symptomatic patients accounted for 32.6% of the development cohort and 31.4% of the independent internal validation cohort. Age was missing in 18 patients (10.4%) and was replaced with the cohort median of 57 years before analysis. Height and weight were excluded from model development because of substantial missingness, and no unresolved missing values remained among the variables entered into the final models.

**Figure 1:**
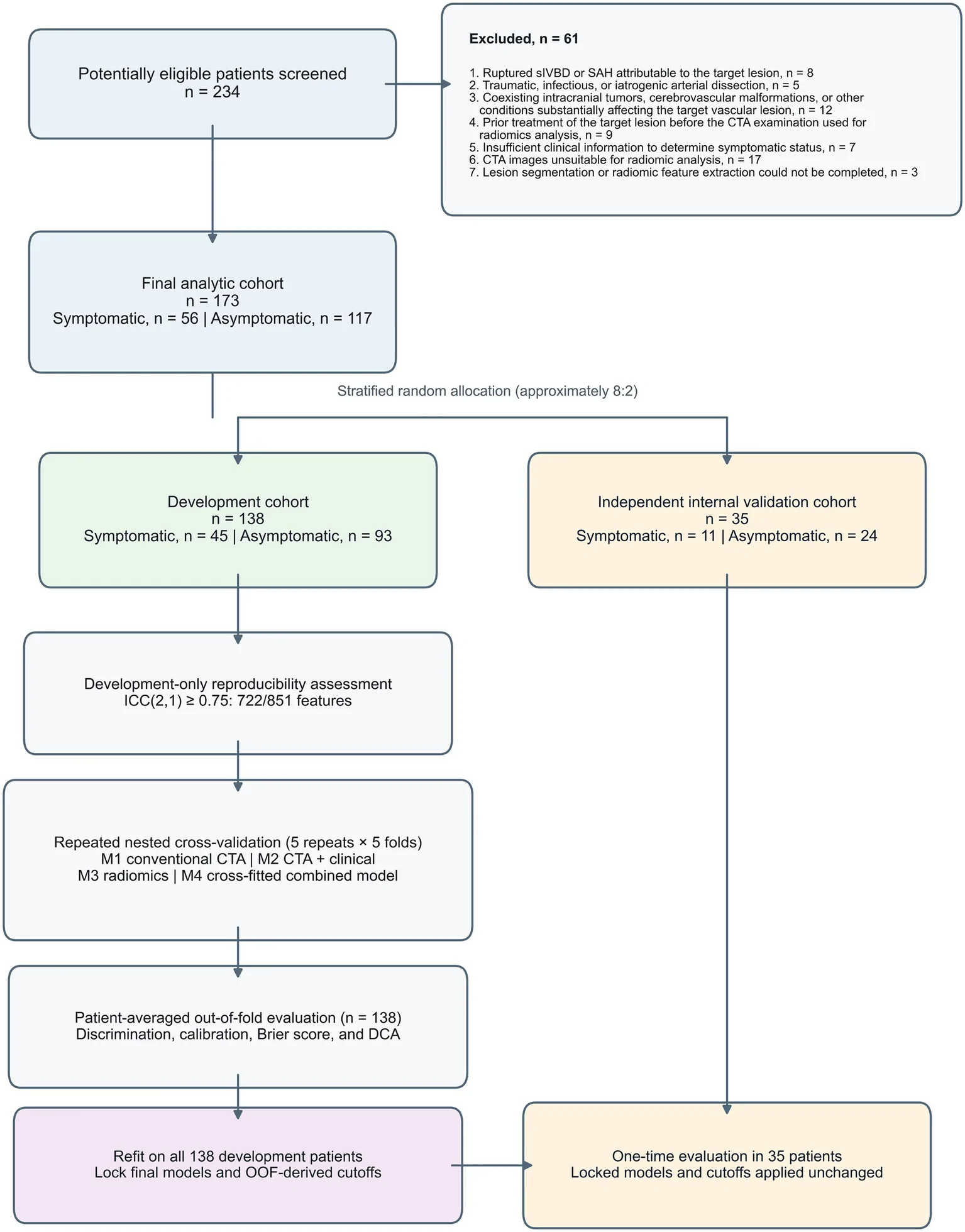
Study flow and analysis workflow. A total of 234 potentially eligible patients were screened, of whom 61 were excluded according to the prespecified eligibility criteria, leaving 173 patients in the final analytic cohort (56 symptomatic and 117 asymptomatic). Patients were stratified by symptomatic status and randomly allocated at an approximately 8:2 ratio to a development cohort (*n* = 138; 45 symptomatic and 93 asymptomatic) and an independent internal validation cohort (*n* = 35; 11 symptomatic and 24 asymptomatic). In the development cohort, radiomic feature reproducibility was assessed using ICC(2,1), and 722 of 851 features met the prespecified threshold of 0.75. Model development and primary performance assessment were conducted using five-times-repeated five-fold nested cross-validation. Final models and development-derived classification cutoffs were subsequently locked and applied without modification to the independent internal validation cohort. CTA, computed tomography angiography; DCA, decision-curve analysis; ICC, intraclass correlation coefficient; OOF, out-of-fold; sIVBD, spontaneous intracranial vertebrobasilar artery dissection.

In the overall cohort, between-group differences were observed in sex distribution (*p* = 0.034), hypertension (73.2% vs. 49.6%, *p* = 0.003), history of cerebral infarction (37.5% vs. 12.0%, *p* < 0.001), modified Rankin Scale score (*p* < 0.001), and lesion location (*p* = 0.002). Symptomatic patients also had greater lesion length (median, 12.55 vs. 8.36 mm; *p* < 0.001) and lesion width (median, 7.06 vs. 4.38 mm; *p* < 0.001). Regarding subsequent treatment, 43 of 56 symptomatic patients (76.8%) underwent endovascular treatment and 13 (23.2%) were managed conservatively, whereas 81 of 117 asymptomatic patients (69.2%) underwent endovascular treatment and 36 (30.8%) were managed conservatively. The distribution of treatment strategies did not differ significantly between the two groups (*p* = 0.302). The remaining baseline characteristics are summarized in Table 1. Baseline characteristics of the development and independent internal validation cohorts are presented in Supplementary Table S1. The largest absolute standardized mean differences were observed for modified Rankin Scale score (0.208), coronary heart disease (0.202), lesion length (0.201), and lesion width (0.192).

**Table 1:** Baseline characteristics of the entire study population by symptomatic status.

Variable	Overall (*n* = 173)	Asymptomatic (*n* = 117)	Symptomatic (*n* = 56)	*p* value
Age	56.61 ± 10.09	56.79 ± 9.68	56.25 ± 10.97	0.902
Sex	42 (24.3%)	34 (29.1%)	8 (14.3%)	0.034
Smoking	36 (20.8%)	22 (18.8%)	14 (25.0%)	0.348
Drinking	18 (10.4%)	9 (7.7%)	9 (16.1%)	0.091
Hypertension	99 (57.2%)	58 (49.6%)	41 (73.2%)	0.003
Diabetes	25 (14.5%)	17 (14.5%)	8 (14.3%)	0.966
Coronary heart disease	16 (9.2%)	9 (7.7%)	7 (12.5%)	0.307
Hyperlipidemia	18 (10.4%)	10 (8.5%)	8 (14.3%)	0.247
Family history	1 (0.6%)	1 (0.9%)	0 (0.0%)	1.000
Cerebral infarction	35 (20.2%)	14 (12.0%)	21 (37.5%)	<0.001
SAH	2 (1.2%)	1 (0.9%)	1 (1.8%)	0.544
mRS	0.00 [0.00, 1.00]	0.00 [0.00, 0.00]	1.00 [0.00, 1.00]	<0.001
lesion location	64 (37.0%)	34 (29.1%)	30 (53.6%)	0.002
Length	9.68 [6.51, 14.00]	8.36 [6.09, 12.30]	12.55 [7.79, 17.80]	<0.001
Width	4.92 [3.45, 7.54]	4.38 [3.24, 7.05]	7.06 [3.89, 9.86]	<0.001
Parent artery stenosis	13 (7.5%)	11 (9.4%)	2 (3.6%)	0.227
Endovascular treatment	124 (71.7%)	81 (69.2%)	43 (76.8%)	0.302

Data are mean ± standard deviation, median [interquartile range], or n (%), as appropriate. *p* values are descriptive and were not used for predictor selection. Continuous variables were compared using two-sided Mann–Whitney U tests; categorical variables used Pearson chi-square or Fisher exact tests as appropriate.

### Radiomic feature reproducibility and model development

Of the 851 extracted radiomic features, 722 met the prespecified reproducibility criterion of ICC(2,1) ≥ 0.75, corresponding to a retention rate of 84.8%. The median ICC was 0.888 (interquartile range, 0.844–0.923).

When the radiomics pipeline was refitted using all 138 patients in the development cohort for the final full-development M3 model, all 722 reproducible features passed quality control. After Mann–Whitney U screening, 300 features remained, and 291 were retained after Spearman correlation filtering. Minimum redundancy maximum relevance subsequently retained the 50 highest-ranked features, after which LASSO selected 20 features at *λ* = 4.0370 (Supplementary Figure S2). This sequence represents the final full-development refit; outcome-informed feature selection was repeated independently within the relevant training partitions during repeated nested cross-validation rather than applying a fixed 20-feature set across all outer folds.

The final 20-feature set consisted predominantly of wavelet-derived texture and first-order features and also included the original shape feature Maximum2DDiameterSlice. The complete feature list, ICC estimates, confidence intervals, and standardized LASSO coefficients are provided in Supplementary Table S2. The final tuning parameters for all four models are reported in Supplementary Table S3. M4 combined the 12 prespecified clinical and conventional CTA predictors with a single cross-fitted radiomics probability generated by M3. The cross-fitting procedure is illustrated in Supplementary Figure S1.

### Model performance and comparisons in the development cohort

Primary performance evaluation was based on one patient-averaged out-of-fold prediction per patient. M1 achieved an AUC of 0.6303 (95% CI, 0.5244–0.7363), M2 an AUC of 0.6810 (95% CI, 0.5841–0.7779), M3 an AUC of 0.6755 (95% CI, 0.5796–0.7714), and M4 an AUC of 0.7226 (95% CI, 0.6300–0.8151) (Figure 2 and Table 2). M4 showed the highest numerical discrimination among the four models.

**Figure 2:**
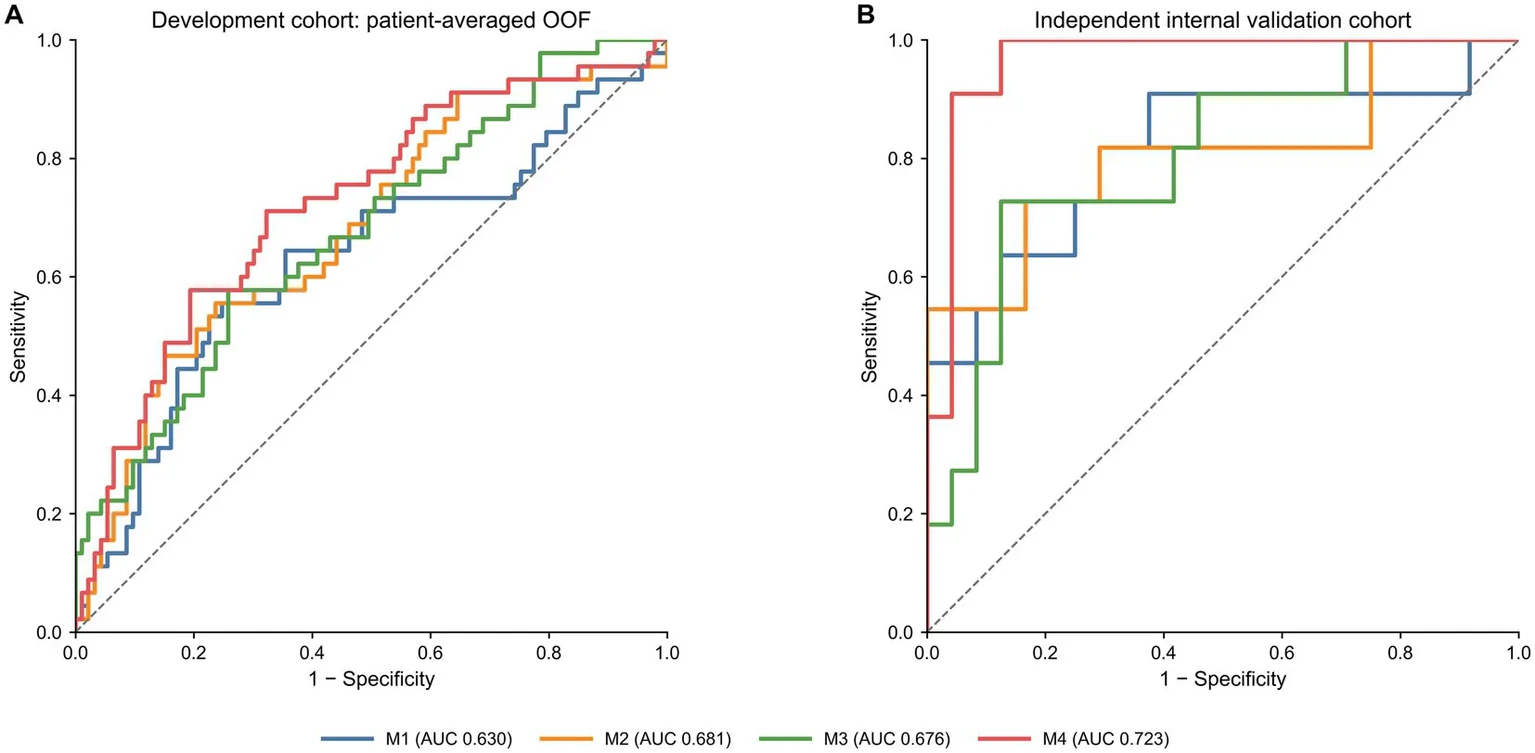
Receiver operating characteristic curves for the four models. **(A)** Receiver operating characteristic curves based on patient-averaged OOF predictions from five-times-repeated five-fold nested cross-validation in the development cohort (*n* = 138). **(B)** Receiver operating characteristic curves obtained after the locked final models were applied to the independent internal validation cohort (*n* = 35). M1 was the conventional CTA model; M2 combined conventional CTA and eligible baseline clinical variables; M3 was the radiomics-only XGBoost model; and M4 combined the predictors in M2 with a cross-fitted radiomics probability generated by M3. AUC values are shown in the figure. AUC, area under the receiver operating characteristic curve; CTA, computed tomography angiography; OOF, out-of-fold; ROC, receiver operating characteristic; XGBoost, extreme gradient boosting.

**Table 2:** Performance of the four models in the development and independent internal validation cohorts.

Cohort	Model	AUC (95% CI)	AP	Brier score
Development, patient-averaged OOF	M1	0.630 (0.524–0.736)	0.476	0.211
Development, patient-averaged OOF	M2	0.681 (0.584–0.778)	0.511	0.203
Development, patient-averaged OOF	M3	0.676 (0.580–0.771)	0.558	0.199
Development, patient-averaged OOF	M4	0.723 (0.630–0.815)	0.559	0.193
Independent internal validation	M1	0.814 (0.638–0.991)	0.768	0.169
Independent internal validation	M2	0.807 (0.624–0.990)	0.782	0.160
Independent internal validation	M3	0.803 (0.641–0.965)	0.688	0.160
Independent internal validation	M4	0.966 (0.909–1.000)	0.914	0.132

OOF denotes out-of-fold; AUC, area under the receiver operating characteristic curve; CI, confidence interval; AP, average precision. Development estimates use one patient-averaged OOF probability per patient.

Average precision values were 0.4756 for M1, 0.5105 for M2, 0.5584 for M3, and 0.5593 for M4. The corresponding Brier scores were 0.2108, 0.2031, 0.1988, and 0.1930, respectively, with M4 yielding the lowest prediction error. Average precision was nearly identical for M3 and M4.

Calibration-in-the-large estimates were close to zero across all models (M1: −0.0068, 95% CI − 0.3796 to 0.3514; M2: 0.0183, 95% CI − 0.3590 to 0.3812; M3: 0.0606, 95% CI − 0.3229 to 0.4300; M4: 0.0138, 95% CI − 0.3670 to 0.3805). Calibration slopes were also close to the ideal value of 1, with estimates of 0.8883 (95% CI 0.1100–1.7169), 0.9701 (95% CI 0.3349–1.6641), 0.9751 (95% CI 0.4448–1.5449), and 1.1887 (95% CI 0.5975–1.8451) for M1–M4, respectively (Figure 3 and Supplementary Table S5).

**Figure 3:**
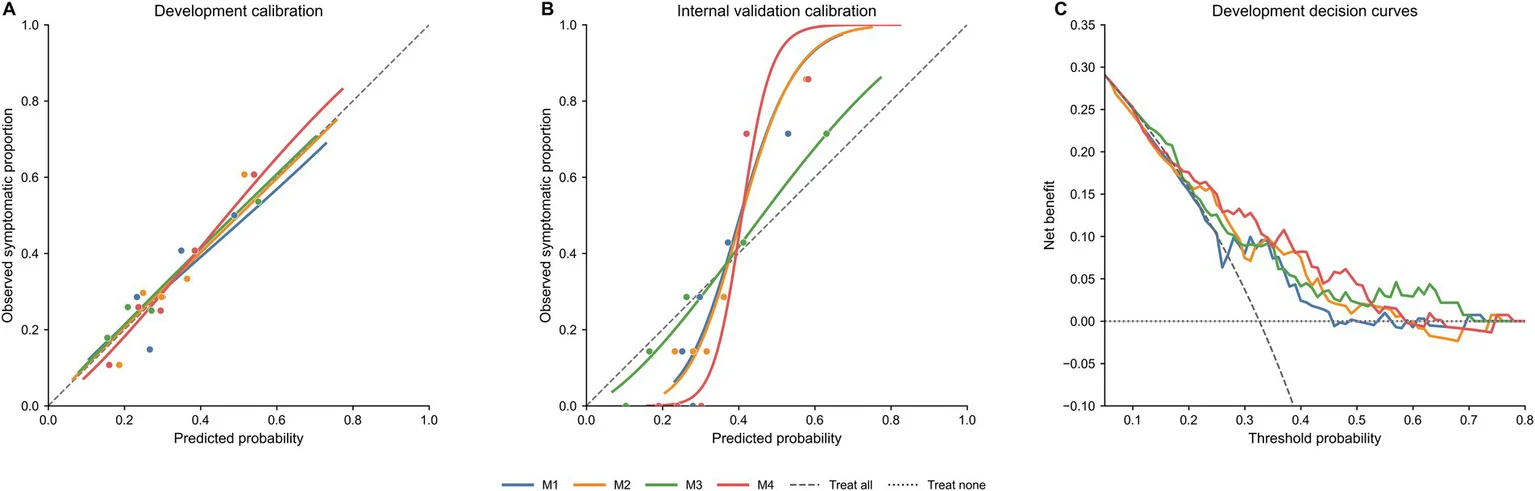
Calibration and exploratory decision-curve analyses of the four models. **(A)** Calibration in the development cohort, evaluated using patient-averaged OOF predictions. **(B)** Calibration after application of the locked models to the independent internal validation cohort. The diagonal dashed line represents perfect agreement between predicted and observed symptomatic proportions; colored points and curves show the observed and model-estimated calibration patterns, respectively. **(C)** Exploratory decision curves in the development cohort. Net benefit is plotted across the evaluated threshold-probability range. The gray dashed and black dotted lines represent the treat-all and treat-none strategies, respectively. M1 was the conventional CTA model; M2 combined conventional CTA and eligible baseline clinical variables; M3 was the radiomics-only XGBoost model; and M4 was the combined model incorporating the predictors in M2 and a cross-fitted radiomics probability. CTA, computed tomography angiography; OOF, out-of-fold; XGBoost, extreme gradient boosting.

In the prespecified primary comparison, M4 showed an AUC increase of 0.0416 relative to M2 (95% CI, −0.0130 to 0.0962; paired DeLong *p* = 0.1357). The Brier score was 0.0101 lower for M4 than for M2, corresponding to a paired difference of −0.0101 (95% bootstrap CI, −0.0208 to 0.0006) (Table 3).

**Table 3:** Prespecified paired model comparisons in the development cohort.

A. Paired AUC comparisons					
Comparison	Role	ΔAUC	95% CI	Raw P	Holm-adjusted P
M4 - M2	Primary	0.0416	−0.0130 to 0.0962	0.136	Not applicable
M2 - M1	Secondary	0.0507	−0.0271 to 0.1284	0.202	0.605
M3 - M1	Secondary	0.0452	−0.1013 to 0.1916	0.546	0.773
M4 - M3	Secondary	0.0471	−0.0595 to 0.1536	0.387	0.773

B. Primary paired brier score comparison			
Comparison	ΔBrier	Bootstrap 95% CI	Replicates
M4 − M2	−0.0101	−0.0208 to 0.0006	2,000

Positive ΔAUC favors the first model. Negative ΔBrier favors M4. Holm adjustment was applied only to the three prespecified secondary AUC comparisons. Inference was based on fixed patient-averaged OOF predictions.

For the secondary AUC comparisons, M2 showed an AUC increase of 0.0507 relative to M1 (raw *p* = 0.2018; Holm-adjusted *p* = 0.6053), M3 showed an increase of 0.0452 relative to M1 (raw *p* = 0.5456; Holm-adjusted *p* = 0.7731), and M4 showed an increase of 0.0471 relative to M3 (raw *p* = 0.3866; Holm-adjusted *p* = 0.7731) (Table 3).

Using the same repeated nested cross-validation partitions and training-specific feature-selection results, GWO-XGBoost achieved an AUC of 0.6755, compared with 0.6504 for Grid-XGBoost. The paired AUC difference was 0.0251 (95% CI, −0.0221 to 0.0723; paired DeLong *p* = 0.2971) (Supplementary Table S4).

In exploratory decision-curve analysis, M4 showed greater net benefit than the treat-all and treat-none strategies across portions of the evaluated threshold range (Figure 3).

### Performance in the independent internal validation cohort

The final locked models were applied without modification to the 35-patient independent internal validation cohort. M1 achieved an AUC of 0.8144 (95% CI, 0.6375–0.9912), M2 an AUC of 0.8068 (95% CI, 0.6239–0.9898), M3 an AUC of 0.8030 (95% CI, 0.6408–0.9653), and M4 an AUC of 0.9659 (95% CI, 0.9093–1.0000) (Figure 2 and Table 2).

The corresponding average precision values were 0.7676, 0.7820, 0.6880, and 0.9136 for M1 through M4, respectively. Brier scores were 0.1694 for M1, 0.1603 for M2, 0.1600 for M3, and 0.1319 for M4. Thus, M4 showed the highest average precision and the lowest Brier score in the independent internal validation cohort.

Calibration-in-the-large estimates were −0.1517 (95% CI, −0.9243 to 0.5575) for M1, −0.1886 (95% CI, −0.9696 to 0.5283) for M2, −0.0033 (95% CI, −0.8246 to 0.7614) for M3, and −0.1638 (95% CI, −0.9569 to 0.5656) for M4. The corresponding calibration slopes were 3.3172 (95% CI, 1.2671 to 6.2159), 3.4322 (95% CI, 1.3666 to 6.5992), 1.3258 (95% CI, 0.4556 to 2.4595), and 6.4466 (95% CI, 2.9194 to 13.4592), respectively (Figure 3 and Supplementary Table S5).

Classification thresholds were determined exclusively from patient-averaged out-of-fold predictions in the development cohort and were applied unchanged to the independent internal validation cohort. At the locked thresholds, sensitivity/specificity were 63.6%/79.2% for M1, 72.7%/79.2% for M2, and 72.7%/83.3% for M3 (Table 4).

**Table 4:** Locked-threshold classification performance in the independent internal validation cohort.

Model	Locked cutoff	TP/FN/TN/FP	Sensitivity, % (95% CI)	Specificity, % (95% CI)	Accuracy, % (95% CI)
M1	0.3421	7/4/19/5	63.636 (35.380–84.834)	79.167 (59.530–90.755)	74.286 (57.931–85.837)
M2	0.3420	8/3/19/5	72.727 (43.435–90.254)	79.167 (59.530–90.755)	77.143 (60.983–87.934)
M3	0.3528	8/3/20/4	72.727 (43.435–90.254)	83.333 (64.147–93.321)	80.000 (64.108–89.958)
M4	0.3167	11/0/19/5	100.000 (74.117–100.000)	79.167 (59.530–90.755)	85.714 (70.624–93.740)

Cutoffs were determined from development OOF predictions and applied unchanged to the independent internal validation cohort. Confidence intervals are Wilson intervals. TP, true positive; FN, false negative; TN, true negative; FP, false positive.

At the locked M4 cutoff of 0.3167, all 11 symptomatic patients were correctly identified, while 19 of 24 asymptomatic patients were correctly classified. This corresponded to a sensitivity of 100.0% (95% CI, 74.1–100.0%), specificity of 79.2% (95% CI, 59.5–90.8%), and accuracy of 85.7% (95% CI, 70.6–93.7%). The positive predictive value was 68.8% (95% CI, 44.4–85.8%), and the negative predictive value was 100.0% (95% CI, 83.2–100.0%) (Table 4 and Supplementary Table S5).

Exploratory decision-curve analysis for the independent internal validation cohort is presented in Supplementary Figure S3.

### Model stability and interpretation

Across the 25 outer evaluation folds, the mean AUCs were 0.6500 for M1, 0.6777 for M2, 0.6646 for M3, and 0.7154 for M4. The corresponding standard deviations were 0.0914, 0.0754, 0.0997, and 0.0618, respectively. M4 had the highest mean outer-fold AUC and the lowest between-fold standard deviation, whereas M3 showed the largest between-fold standard deviation (Figure 4 and Supplementary Table S6).

**Figure 4:**
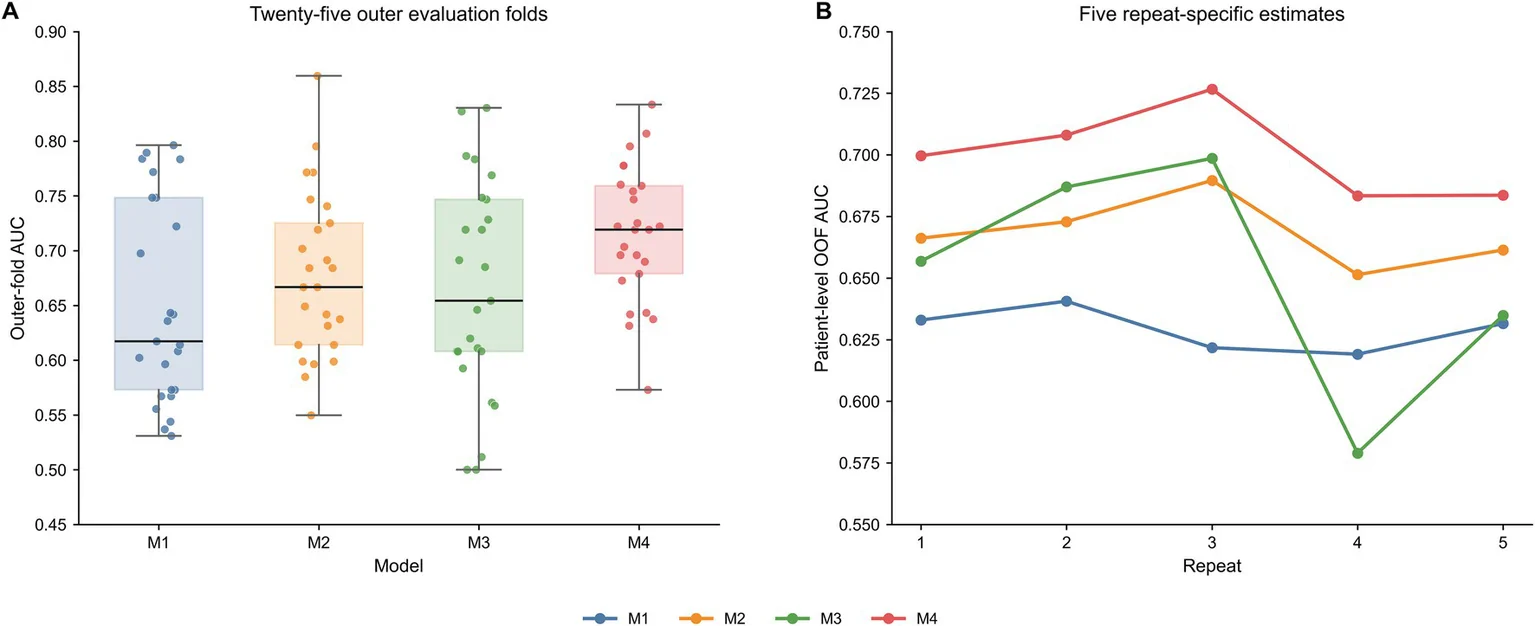
Performance variability across repeated nested cross-validation in the development cohort. **(A)** Distribution of ROC-AUC values across the 25 outer evaluation folds generated by five repeats of five-fold nested cross-validation. Points represent individual outer-fold estimates; boxes summarize their distributions. **(B)** Patient-level OOF AUC for each of the five repeats. M1 was the conventional CTA model; M2 combined conventional CTA and eligible baseline clinical variables; M3 was the radiomics-only XGBoost model; and M4 was the cross-fitted combined model. AUC, area under the receiver operating characteristic curve; CTA, computed tomography angiography; OOF, out-of-fold; ROC, receiver operating characteristic; XGBoost, extreme gradient boosting.

At the repeat level, M4 AUCs ranged from 0.6834 to 0.7266, whereas M3 AUCs ranged from 0.5790 to 0.6986. At the patient level, the median within-patient standard deviation of the five out-of-fold probabilities was 0.0602 for M4 compared with 0.0969 for M3. Complete fold-level, repeat-level, and patient-level results for all four models are provided in Supplementary Table S6.

Radiomic feature selection varied across the 25 outer-training partitions. No individual feature was selected in all 25 partitions. The most frequently retained feature was wavelet-HHH.glszm.ZoneVariance, which was selected in 17 of 25 partitions (68%). This was followed by wavelet-HHL.glszm.ZoneVariance in 16 partitions (64%), wavelet-HHH.firstorder.Skewness and wavelet-LHH.glszm.SizeZoneNonUniformityNormalized in 15 partitions each (60%), and wavelet-LLL.gldm.LargeDependenceLowGrayLevelEmphasis in 13 partitions (52%) (Supplementary Figure S4 and Supplementary Table S7). The mean pairwise Jaccard similarity between outer-partition feature sets was 0.1493, with a median of 0.1364.

In the final M3 model fitted using all 138 development patients, SHAP analysis identified wavelet-HHH.glszm.ZoneVariance as the leading contributor to model predictions, with a mean absolute SHAP value of 0.4481. This was followed by wavelet-LLL.gldm.LargeDependenceLowGrayLevelEmphasis (0.3164) and wavelet-LHH.glszm.SizeZoneNonUniformityNormalized (0.1832) (Figure 5 and Supplementary Table S7). SHAP dependence plots showed apparent nonlinear contribution patterns for the three highest-ranked features (Supplementary Figure S6). A nomogram representation of the final M4 ridge logistic model is provided in Supplementary Figure S5.

**Figure 5:**
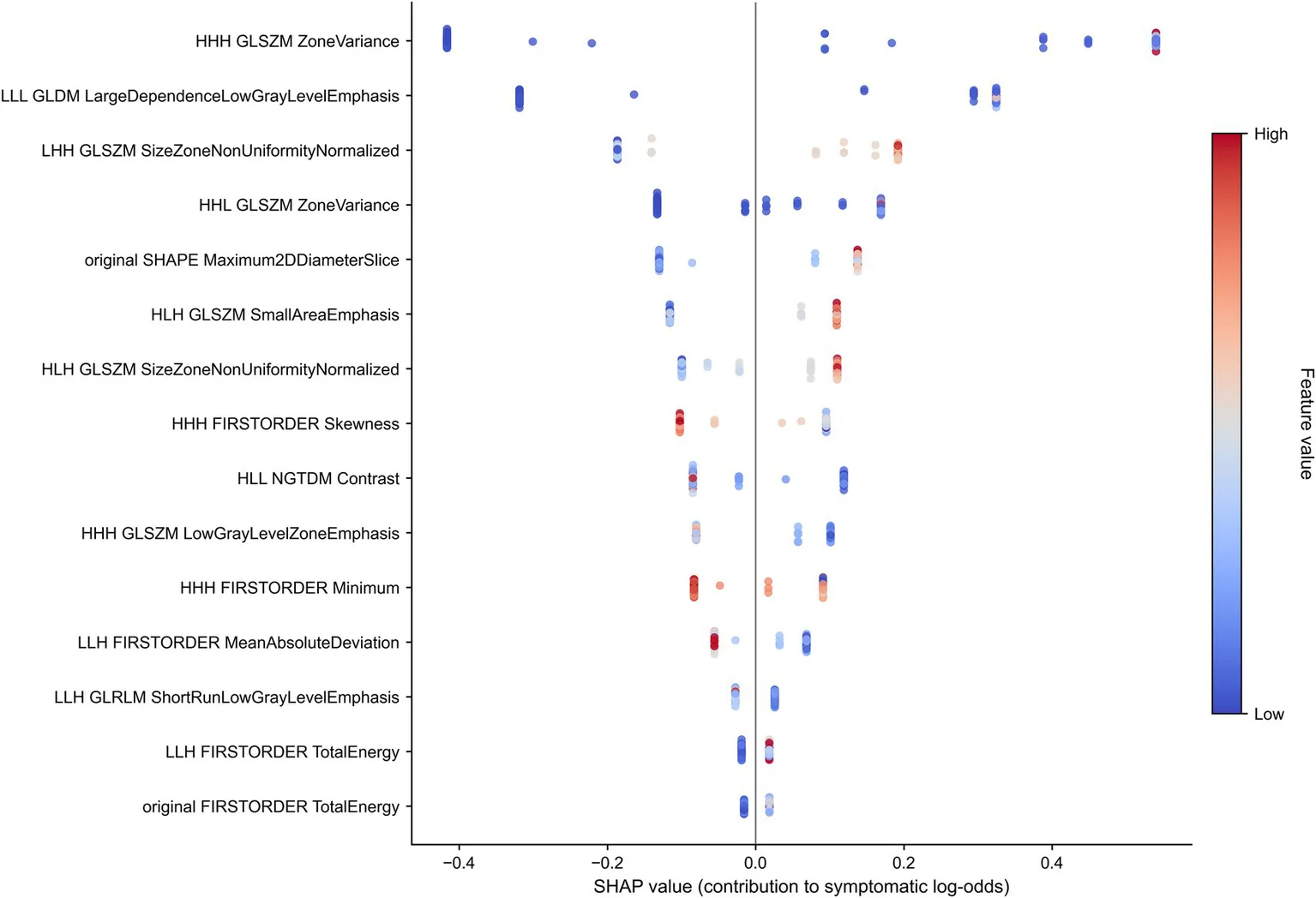
SHAP summary plot for the final full-development radiomics model. The plot shows the 15 radiomic features with the largest mean absolute SHAP values in the final M3 model fitted using all 138 patients in the development cohort. Each point represents one patient. The horizontal position indicates the SHAP contribution to the model output on the symptomatic log-odds scale, with positive values increasing and negative values decreasing the model-predicted log odds of symptomatic status. Point color indicates the corresponding feature value, from low (blue) to high (red). Features are ordered by decreasing mean absolute SHAP value. GLDM, gray-level dependence matrix; GLCM, gray-level co-occurrence matrix; GLRLM, gray-level run-length matrix; GLSZM, gray-level size-zone matrix; NGTDM, neighboring gray-tone difference matrix; SHAP, SHapley Additive exPlanations.

## Discussion

In this study, we developed and internally validated CTA-based models for differentiating symptomatic from asymptomatic unruptured sIVBD using a leakage-controlled analytical framework. Among the four prespecified models, the combined model incorporating conventional CTA findings, eligible clinical variables, and a cross-fitted radiomics probability (M4) showed the highest numerical discrimination in the development cohort, with a patient-averaged out-of-fold AUC of 0.723, and also yielded the lowest Brier score. Relative to the conventional CTA-plus-clinical model (M2), M4 increased the AUC by 0.0416 and reduced the Brier score by 0.0101. In the independent internal validation cohort, M4 yielded the highest numerical AUC and average precision and the lowest Brier score among the four models; however, its markedly elevated calibration slope indicated that these discrimination results did not translate into reliable individual probability estimates. Repeated nested cross-validation showed that M4 had the highest mean outer-fold AUC and the lowest between-fold variability, whereas the radiomics-only model showed greater variability across data partitions. Together, these findings support further evaluation of CTA radiomics as a complementary quantitative imaging component for characterizing symptomatic status in sIVBD.

The incremental contribution of radiomics is particularly relevant because conventional CTA already provides clinically important information on lesion morphology. In the present study, the conventional CTA model (M1) showed modest discrimination, and the addition of eligible baseline clinical variables in M2 was accompanied by a numerical improvement in AUC. The radiomics-only model (M3) achieved discrimination similar to that of M2, while the combined M4 model yielded the highest overall performance. This pattern suggests that radiomics may capture information that is not fully represented by routinely recorded clinical characteristics or conventional CTA measurements (15, 16). The formal comparison between M4 and M2 showed an AUC increase of 0.0416, although the 95% confidence interval included zero and the paired DeLong *p* value was 0.1357. The corresponding Brier score difference showed a similar direction of improvement. Thus, the observed results are consistent with a possible incremental contribution from radiomics, but the magnitude and reproducibility of this contribution require confirmation in larger datasets. Importantly, this should not be interpreted as evidence that radiomics replaces conventional CTA assessment; rather, its potential value lies in complementing information that is already available in routine imaging and clinical evaluation.

The comparison between radiomics and conventional morphology provides further context for this interpretation. M3 showed a numerically higher AUC than M1, but the paired difference was imprecisely estimated. At the feature level, original.shape.Maximum2DDiameterSlice showed minimal correlation with manually recorded lesion length and width, indicating that this PyRadiomics-derived shape descriptor was not directly interchangeable with either conventional size measurement in the present dataset. This observation suggests that radiomics may provide a mathematically distinct representation of lesion geometry in addition to higher-order texture information. Nevertheless, conventional measurements such as lesion location, length, width, and parent-artery stenosis remain clinically interpretable and immediately available, and the current findings support integration rather than substitution of conventional and radiomic information (15, 17).

The optimization analysis also warrants a measured interpretation. GWO-XGBoost achieved a numerically higher patient-averaged out-of-fold AUC than Grid-XGBoost under the implemented nested cross-validation framework. In addition, GWO and Grid Search were implemented using different search spaces and computational budgets. The present comparison therefore reflects the performance of the two specific tuning strategies used in this study rather than a matched-compute comparison of the optimization algorithms themselves. GWO provided a feasible approach for exploring a multidimensional XGBoost hyperparameter space (13), but the current results do not establish its intrinsic superiority over Grid Search. This distinction is important because hyperparameter-search performance may depend on the search space, evaluation budget, random initialization, and upstream feature-selection procedure as well as on the optimizer itself.

Model interpretation and stability analyses provided additional insight into the behavior of the radiomics pipeline. In the final M3 model, the largest mean absolute SHAP contributions were observed for wavelet-HHH.glszm.ZoneVariance, wavelet-LLL.gldm.LargeDependenceLowGrayLevelEmphasis, and wavelet-LHH.glszm.SizeZoneNonUniformityNormalized. These features quantify aspects of gray-level organization and spatial heterogeneity within the segmented lesion and therefore characterize image information beyond simple geometric measurements. However, SHA*p* values describe how the fitted model uses individual features to modify its predictions; they should not be interpreted as evidence that these radiomic features are causal determinants of symptomatic presentation (14, 18). Likewise, specific biological interpretations linking individual wavelet-derived features directly to intramural hematoma, thrombus, or vessel-wall instability cannot be established from the present data alone and would require validation against dedicated pathological or vessel-wall imaging reference standards.

The repeated nested cross-validation results also highlighted a distinction between model-level stability and feature-level stability. M4 had the highest mean outer-fold AUC and the lowest between-fold standard deviation among the four models, while the within-patient variability of repeated out-of-fold probabilities was lower for M4 than for M3. These findings suggest that integrating conventional CTA and clinical information with the radiomics probability may reduce the dependence of individual predictions on a particular data partition. In contrast, the composition of the radiomic feature set varied substantially across outer-training partitions. No individual feature was selected in all 25 partitions, and the mean pairwise Jaccard similarity between selected feature sets was low. This pattern is plausible in a high-dimensional setting in which multiple correlated radiomic features may encode overlapping information and can substitute for one another across resampled training sets (18). Accordingly, the present findings provide stronger support for interpretation at the model level than for assigning biological significance to any single radiomic feature.

The independent internal validation cohort provided a separate assessment after all feature-selection, model-fitting, hyperparameter-optimization, and threshold-selection procedures had been completed using the development cohort. M4 yielded an AUC of 0.966 in this cohort and, at the locked development-derived cutoff, identified all 11 symptomatic patients while correctly classifying 19 of 24 asymptomatic patients. Nevertheless, the validation AUC should not be interpreted as evidence that the model performed better than in the development cohort. AUC evaluates the ranking of patients rather than the accuracy of their predicted probabilities and can remain high in the presence of substantial miscalibration.

For M4, the validation calibration slope was 6.4466 (95% CI, 2.9194–13.4592), indicating that its predicted log odds were insufficiently dispersed. The jointly estimated calibration intercept was 2.3833 (95% CI, 0.5519–5.1424), whereas the calibration-in-the-large was −0.1638 (95% CI, −0.9569 to 0.5656). Although the mean predicted probability was close to the observed symptomatic proportion, this agreement at the cohort level did not establish reliable calibration for individual patients. The difference between the development and validation AUCs, together with the imprecisely estimated calibration parameters, may reflect sampling variability and case-mix characteristics in a validation cohort containing only 35 patients. These validation results should therefore be regarded as preliminary, and the predicted probabilities should not be used directly for individual clinical decision-making without evaluation in a larger external cohort and, if necessary, recalibration (19).

From a clinical perspective, CTA radiomics has the practical advantage of deriving quantitative information from an imaging examination that is already routinely available in patients with suspected sIVBD. The present results suggest that radiomic analysis may complement conventional visual and morphologic assessment by providing reproducible numerical descriptors of lesion geometry and image heterogeneity (15, 16). The exploratory decision-curve analyses also showed favorable net-benefit patterns for M4 over portions of the evaluated threshold range (20). These findings support further evaluation of CTA radiomics as an adjunctive tool for cross-sectional characterization of symptomatic status. However, the endpoint evaluated in this study was symptomatic versus asymptomatic status at the time of clinical assessment. The models were not developed to predict subsequent enlargement, rupture, future symptom development, or treatment response. Accordingly, their potential role should currently be considered supportive rather than prescriptive, and application to treatment selection would require prospective evaluation within a clearly defined clinical decision pathway.

Several limitations should be acknowledged. First, this was a retrospective, single-center study with 173 patients. Although repeated nested cross-validation reduced dependence on a single development split, the development cohort contained only 45 symptomatic patients, limiting the precision of comparisons between models. The independent internal validation cohort included only 35 patients, with 11 symptomatic events. Consequently, both discrimination and calibration estimates were sensitive to sampling variability. The apparently higher validation AUC may reflect the particular case mix of this small cohort, while the markedly elevated and imprecisely estimated calibration slope limits interpretation of the predicted probabilities.

Second, lesion segmentation required manual refinement. Although feature-level inter-reader reproducibility was assessed using three independent segmentations and 84.8% of extracted radiomic features met the prespecified ICC criterion, manual segmentation remains a potential source of measurement variability (21, 22). Third, the ICC reproducibility filter was determined once using the full development cohort before repeated nested cross-validation. This step was outcome-independent because it did not use symptomatic-status labels, whereas all subsequent outcome-informed selection and tuning procedures were restricted to the relevant training data; nevertheless, this shared preprocessing step should be considered when interpreting the validation framework. Fourth, treatment allocation was not randomized and may have been influenced by symptomatic status, lesion morphology, and physician judgment. Although treatment strategy was not used as an inclusion or exclusion criterion, the distribution of neurosurgical, endovascular, and conservative management may reflect underlying differences in disease presentation and could therefore introduce residual treatment-related selection bias. Finally, decision-curve analysis was exploratory because no prespecified clinical action threshold was available, and the cross-sectional design precludes inference regarding future lesion progression, rupture, symptom development, or treatment benefit. Larger multicenter studies with true external validation, prospective assessment, and predefined clinical decision pathways are needed to address these limitations (23).

## Conclusion

In this single-center retrospective study, a combined model integrating conventional CTA features, clinical variables, and a cross-fitted radiomics probability showed the best overall performance for distinguishing symptomatic from asymptomatic unruptured sIVBD. The findings suggest that CTA radiomics may provide complementary quantitative information beyond conventional clinical and morphological assessment.

## Data Availability

The original contributions presented in the study are included in the article/Supplementary material, further inquiries can be directed to the corresponding authors.

## Supplementary material

SUPPLEMENTARY FIGURE S1 Cross-fitting workflow for development and evaluation of the combined model M4. Within each outer partition, patients were separated into training patients (T) and held-out evaluation patients (E). To select the ridge regularization parameter for M4, T was divided into five internal training-validation splits, denoted A and B. Within A, the complete M3 radiomics pipeline was cross-fitted to generate radiomics probabilities for M4 training; a separate M3 model fitted using all patients in A generated probabilities for B without using outcomes from B in upstream development. These radiomics probabilities were combined with the 12 conventional CTA and eligible clinical predictors, and the M4 ridge parameter C was selected by mean validation log loss. After selection, cross-fitted M3 probabilities were regenerated for all patients in T to fit M4, while an M3 model fitted using all of T generated radiomics probabilities for E. The fitted M4 model was then applied to E to obtain the outer-fold prediction. The same leakage-controlled cross-fitting logic was used when M4 was refitted using all 138 development patients. CTA, computed tomography angiography; M3, radiomics-only XGBoost model; M4, combined ridge logistic-regression model; XGBoost, extreme gradient boosting.

SUPPLEMENTARY FIGURE S2 Radiomic feature reproducibility and selection in the final full-development model. **(A)** Distribution of ICC(2,1) estimates for the 851 extracted radiomic features. The red dashed line indicates the prespecified retention threshold of 0.75, and the black dotted line indicates the median ICC of 0.888. **(B)** Number of features retained at each stage when the radiomics pipeline was refitted using all 138 development patients. Of 851 extracted features, 722 passed the ICC filter and quality control, 300 remained after Mann-Whitney U screening, 291 after Spearman correlation filtering, 50 after minimum redundancy maximum relevance selection, and 20 after LASSO selection. Outcome-informed selection was repeated independently within the relevant training partitions during nested cross-validation; the sequence shown in panel B represents the final refit using the complete development cohort. ICC, intraclass correlation coefficient; LASSO, least absolute shrinkage and selection operator; mRMR, minimum redundancy maximum relevance; MWU, Mann-Whitney U test.

SUPPLEMENTARY FIGURE S3 Exploratory decision-curve analysis in the independent internal validation cohort. Net benefit is shown across the evaluated threshold-probability range after the four locked models were applied to the independent internal validation cohort (*n* = 35). The gray dashed and black dotted lines represent the treat-all and treat-none strategies, respectively. M1 was the conventional CTA model; M2 combined conventional CTA and eligible baseline clinical variables; M3 was the radiomics-only XGBoost model; and M4 combined the predictors in M2 with the radiomics probability generated by the final M3 model. CTA, computed tomography angiography; XGBoost, extreme gradient boosting.

SUPPLEMENTARY FIGURE S4 Stability of radiomic feature selection across repeated nested cross-validation. **(A)** Selection frequencies of radiomic features retained in at least one of the 25 outer-training partitions. Bar labels give the number of partitions in which each feature was selected; features are ordered by decreasing frequency. **(B)** Heat map of pairwise Jaccard similarity between the feature sets selected in the 25 outer-training partitions. Partition labels identify the repeat and fold (R1F1-R5F5), and darker colors indicate greater overlap. The diagonal represents self-comparisons. GLDM, gray-level dependence matrix; GLRLM, gray-level run-length matrix; GLSZM, gray-level size-zone matrix; NGTDM, neighboring gray-tone difference matrix.

SUPPLEMENTARY FIGURE S5 Nomogram representation of the final combined model M4. **(A)** Nomogram for estimating the probability of symptomatic status from lesion location, lesion length, maximum width, parent-artery stenosis, age, sex, smoking, alcohol consumption, hypertension, diabetes mellitus, coronary artery disease, dyslipidemia, and the radiomics probability generated by M3. For each predictor, the corresponding point value is read from the upper scale; the sum of all points is mapped to the symptomatic-probability scale. Binary predictors use the 0/1 coding specified in Supplementary Table S3. **(B)** Expanded display of the signed point change contributed by each clinical or conventional CTA predictor across its observed range or from category 0 to category 1. Positive values increase and negative values decrease the model-predicted log odds of symptomatic status. The nomogram visualizes the final fitted equation and does not represent a separately trained model. CTA, computed tomography angiography; M3, radiomics-only XGBoost model; M4, combined ridge logistic-regression model; XGBoost, extreme gradient boosting.

SUPPLEMENTARY FIGURE S6 SHAP dependence plots and representative patient-level explanations for the final M3 radiomics model. **(A–C)** SHAP dependence plots for the three radiomic features with the largest mean absolute SHAP values: wavelet-HHH GLSZM ZoneVariance, wavelet-LLL GLDM LargeDependenceLowGrayLevelEmphasis, and wavelet-LHH GLSZM SizeZoneNonUniformityNormalized, respectively. Each point represents one patient in the development cohort; the vertical axis shows the feature contribution on the model log-odds scale. **(D)** SHAP contributions for the patient with the lowest M3-predicted probability (patient ID 25; predicted probability, 0.061). **(E)** SHAP contributions for the patient with the highest M3-predicted probability (patient ID 137; predicted probability, 0.778). Negative contributions decrease and positive contributions increase the predicted log odds of symptomatic status; contributions from features not individually displayed are combined as “Other features.” GLDM, gray-level dependence matrix; GLSZM, gray-level size-zone matrix; M3, radiomics-only XGBoost model; SHAP, SHapley Additive exPlanations; XGBoost, extreme gradient boosting.

## Glossary

sIVBD: spontaneous intracranial vertebrobasilar artery dissection
SAH: subarachnoid hemorrhage
CTA: computed tomography angiography
DSA: digital subtraction angiography
ROI: region of interest
GLCM: gray-level co-occurrence matrix
GLSZM: gray-level size zone matrix
GLRLM: gray-level run length matrix
GLDM: gray-level dependence matrix
NGTDM: neighboring gray tone difference matrix
LoG: laplacian of gaussian
LBP: local binary pattern
mRMR: minimum redundancy maximum relevance
AUC: area under the curve
ROC: receiver operating characteristic
DCA: Decision curve analysis
XGBoost: eXtreme Gradient Boosting
GWO: Grey Wolf Optimizer
SHAP: SHapley Additive exPlanations
mRS: modified Rankin Scale
MID: mutual information difference
NPV: negative predictive values
PPV: positive predictive values
