# Supplementary material for: Development and internal validation of CTA-based radiomics models for differentiating symptomatic from asymptomatic unruptured spontaneous intracranial vertebrobasilar artery dissection
Source: Front Neurol. 2026 Oct 23;17:1883223. doi: 10.3389/fneur.2026.1883223 (PMC13739547; doi:10.3389/fneur.2026.1883223)
Supplement: Supplementary file 2 [file Data_Sheet_2.ZIP › Supplementary methods/Supplementary_methods(1).docx]

**Supplementary Methods**

**S1 Reproducibility and Radiomic Feature Selection**

**Reproducibility assessment**

ICC was calculated from the three reader-specific feature measurements in the development cohort using ICC(2,1), a two-way random-effects, absolute-agreement, single-measurement model. Retention required an ICC point estimate ≥ 0.75. The 95% confidence interval for each ICC estimate was calculated using the ANOVA-based F-distribution method. Feature retention was based on the ICC point estimate rather than the lower bound of its 95% confidence interval.

This outcome-independent filter was established using the development cohort before nested modeling and was shared across subsequent development partitions. It was not recalculated within each fold. Reader 1 feature values were used for subsequent screening, fitting, and prediction. ICC assessed feature-value reproducibility; spatial overlap between reader-specific ROIs was not evaluated using the Dice similarity coefficient.

**Quality control and imputation**

Within each training subset, features with more than 20% non-finite values were excluded. Remaining non-finite values were replaced by the training-subset median. Features with only one unique value or with one value accounting for at least 95% of observations after imputation were removed.

Feature eligibility and imputation parameters were estimated from training data and applied unchanged to the corresponding evaluation subset.

**Univariate screening and correlation filtering**

A two-sided Mann–Whitney *U* test compared training feature values between outcome groups. Features with *P* < 0.05 were retained. This threshold was a screening rule and did not constitute multiplicity-adjusted evidence for individual biomarkers.

Candidates were ordered by increasing *P* value, decreasing absolute rank-biserial correlation, and alphabetical feature name. Spearman correlations were calculated from imputed training values. Candidates were considered sequentially and retained only if their absolute correlation with every previously retained feature was ≤ 0.90. Thus, a feature was excluded when at least one previously retained feature had |*ρ*| > 0.90.

**mRMR selection**

mRMR used the mutual information difference criterion. Candidate features were discretized into up to 10 quantile-based bins using ordinal encoding, linear quantile interpolation, and no subsampling. Degenerate boundaries could reduce the realized number of bins.

Relevance was the mutual information between a candidate and the outcome. Redundancy was its mean mutual information with previously selected features, with redundancy set to zero for the first selection. At each step, the candidate maximizing relevance minus redundancy was retained. Ties were resolved by higher relevance and then alphabetical feature name. Selection continued until 50 candidates were retained or the available set was exhausted.

**LASSO selection**

LASSO logistic regression evaluated 100 logarithmically spaced *λ* values from 10^−4^ to 10^2^, implemented as *C* = 1/*λ* in scikit-learn. Shuffled stratified five-fold cross-validation selected *λ*. Within each fold, median imputation and z-score standardization were fitted on its training portion and applied to its validation portion.

For each value of *λ*, the binomial deviance in each validation fold was calculated as −2 times the total binomial log-likelihood, and the five fold-specific deviances were then averaged. The value of *λ* that minimized this mean was selected. Exact ties favored the first value in the ascending *λ* grid, corresponding to the smaller value of *λ*.

The preceding univariate screening, correlation filtering, and mRMR steps used the parent training subset; they were not repeated within each fold used solely to select λ. They were repeated within the training partitions used for upstream radiomics hyperparameter evaluation and held-out prediction.

The cross-validation coefficient path used the SAGA solver, a maximum of 5,000 iterations, and a tolerance of 10^−4^. Final LASSO refitting on the complete parent training subset used a maximum of 20,000 iterations and a tolerance of 10^−6^. Features with absolute standardized coefficients > 10^−8^ were retained. Iteration counts and iteration-limit indicators were recorded.

The selected feature columns were passed to XGBoost on their original scales with training-derived median imputation. If no usable feature remained, the corresponding radiomics prediction used the training-subset outcome prevalence. No manual target for the final selected-feature count was imposed.

**S2 Model Optimization and Randomness Control**

**Ridge logistic regression**

M1, M2, and M4 used L_2_-penalized logistic regression with an intercept. Age, lesion length, maximum width, and radiomics probability were treated as continuous predictors where applicable. Missing values were replaced by training medians, and predictors were centered and scaled using training means and population standard deviations. Continuous predictors with zero training variance were transformed to zero.

Binary predictors retained 0/1 coding. Missing values were replaced by the training mode, with ties resolved as 0. Parent artery stenosis was coded as 0 for absence and 1 for presence.

The regularization parameter *C* was selected from 100 logarithmically spaced values between 10^−4^ and 10^4^. The selection criterion was the mean log loss across the five inner-validation folds. Preprocessing was fitted within each inner-training subset. Ties favored the first value in the ascending *C* grid, corresponding to stronger regularization. Fits used LBFGS, a maximum of 5,000 iterations, and a tolerance of 10^−9^.

**GWO-XGBoost**

XGBoost was fitted using objective = "binary:logistic" and eval_metric = "auc", with nthread = 1. Candidate fitness was assessed using ROC-AUC.

The GWO search ranges were learning_rate = 0.01–0.30; max_depth = 2–10; min_child_weight = 1–10; subsample = 0.50–1.00; colsample_bytree = 0.50–1.00; gamma = 0–5; reg_alpha = 0–5; reg_lambda = 0–5; and n_estimators = 100–800.

Wolf coordinates were represented in [0, 1], clipped to these bounds, and linearly mapped to the parameter ranges. max_depth, min_child_weight, and n_estimators were rounded to integer values using Python’s built-in rounding rule. Each run used 25 wolves and 60 fixed iterations, giving 1,500 candidate evaluations; decoded configurations could recur.

Fitness was the arithmetic mean AUC across five inner-validation folds. Each fold used features selected exclusively from its inner-training subset, with those selections reused across hyperparameter candidates. The three leader solutions were updated sequentially using strict fitness improvements. The exploration coefficient followed *a*(*t*) = 2 − 2*t*/60 for *t* = 0, 1, …, 59. Wolf positions were updated from their three leader-based positions and clipped to [0, 1]. Optimization terminated after 60 iterations without convergence-based early stopping.

After optimization, feature selection was performed on the complete corresponding training subset, and XGBoost was fitted using the selected configuration and tree count.

**Grid-XGBoost**

Grid Search evaluated the following candidate sets: learning_rate ∈ {0.03, 0.10, 0.20}; max_depth ∈ {3, 5, 7}; min_child_weight ∈ {1, 3, 5}; subsample ∈ {0.70, 1.00}; colsample_bytree ∈ {0.70, 1.00}; gamma ∈ {0, 1, 3}; reg_alpha ∈ {0, 0.5, 1.0}; reg_lambda ∈ {1, 3, 5}; and n_estimators ∈ {200, 400, 600}. The Cartesian product contained 8,748 configurations per outer partition.

Outer and inner partitions and inner-training feature selections were identical to those used for GWO. For computational efficiency, each of the 2,916 combinations of the other eight parameters was fitted to 600 boosting rounds per inner fold, and predictions using the first 200, 400, and 600 trees were evaluated. Consequently, 8,748 configuration evaluations did not require 8,748 independently trained boosting paths per fold.

The configuration with the highest mean inner AUC was selected, with ties resolved by the first configuration in the predefined enumeration. It was refitted for the selected number of trees on the outer training subset, using the same final feature selection as the corresponding GWO model. The comparison therefore concerned these implemented search strategies, which differed in search spaces, evaluation budgets, and computation reuse.

**Random seeds**

The master seed was 42, and the prespecified cohort and outer-fold assignments were retained throughout the analysis. For outer-partition index *k* = 0, 1, …, 24, the modeling seed was defined as *s* = 42 + 10,000*k*. Inner partitions used *s* + 101; inner feature selection used *s* + 1,000 + *j* for *j* = 0, 1, …, 4; GWO initialization used *g* = *s* + 2,000; final feature selection used *s* + 4,000; and final GWO-XGBoost fitting used *s* + 7,000.

At GWO iteration *t* = 0, 1, …, 59, wolf *i* = 0, 1, …, 24 used evaluation seed *g* + 100,000 + 1,000*t* + 31*i*, with an additional 17*j* for its inner-fold fit. LASSO coefficient-path fits used the selection seed plus their fold index, and final LASSO refitting used the selection seed. Grid inner fits used *s* + 703,000 + 17*j*, and final Grid fitting used *s* + 8,000.

For the final radiomics model fitted using all 138 development patients, the seeds were 900,143 for inner partitions, 901,042 + *j* for inner feature selection, 902,042 for GWO, 42 for final feature selection, and 940,042 for final XGBoost fitting.

Additional M4 contexts used a deterministic function *H*: the list [42, context arguments, …] was serialized using Python JSON with sorted keys and string conversion for unsupported values, hashed using SHA-256, and the first eight hexadecimal characters were converted to an integer modulo 2^31^ − 1.

For training seed *s* and internal-fold index *j*, the label was constructed using the template “M4_<s>_meta<j>”. Cross-fitting partitions used H(label, "crossfit_folds"); upstream cross-fitting model seeds used H(label, "crossfit_model", fold index); and the model fitted on the complete inner-training subset used H(label, "full_A"). Final M4 cross-fitting partitions used H(s, "M4_final_crossfit_folds"), with upstream labels constructed using the template “M4_<s>_final”. The final M4 model fitted using the entire development cohort used seed 900,042. Each upstream radiomics model then used the applicable partition and optimization offsets.

**S3 Cross-Fitted Development of M4**

For an outer partition, let T denote training patients and E denote held-out evaluation patients. All outcome-informed development for that partition used T. M3 was developed within T and generated radiomics probabilities for E.

To select the M4 regularization parameter, *T* was divided into five inner training–validation splits. For one such split, let *A* denote the inner-training patients and *B* the inner-validation patients.

A was further divided into five cross-fitting folds. For each held-out portion of A, a complete upstream radiomics model was developed using the remaining portions, including feature screening, LASSO, GWO optimization, and model fitting. This generated one radiomics probability per patient in A from a model that had not used that patient’s outcome.

A separate radiomics model was developed using all patients in A and was used to predict B. Therefore, the outcomes in B did not contribute to the upstream procedures that generated either the cross-fitted training probabilities in A or the inner-validation probabilities in B. Ridge models combined the 12 clinical and CTA predictors with these probabilities, were fitted using A, and were evaluated by log loss in B. The regularization parameter C was selected according to the mean validation log loss across the five inner splits.

After selecting C, fresh five-fold cross-fitted probabilities were generated for all of T. These were combined with the 12 baseline predictors to fit the final M4 ridge model on T. For E, probabilities from M3 fitted on all of T were entered into the fitted M4 model.

The same procedure was applied when fitting M4 using all 138 patients in the development cohort. For patients in the independent internal validation cohort, radiomics probabilities were first generated by the final M3 model and were then combined with the baseline predictors in the final M4 model. Thus, model training used cross-fitted upstream probabilities, whereas prediction in the independent internal validation cohort used probabilities generated by the upstream model fitted using the entire development cohort.

The combined model was:

$$P(Y = 1) = \frac{1}{1 + exp(-\eta)},$$

with

$$\eta= \beta_{0} + \beta_{\mathrm{rad}}p_{\mathrm{rad}} + \sum_{j = 1}^{12} \beta_{j}X_{j}.$$

Here, *p*_rad_ denotes the probability generated by the XGBoost radiomics model, and *X_j_* denotes the *j*th clinical or conventional CTA predictor. The nomogram represented this fitted equation and was not a separately trained model.

Patient identifiers were retained for each partition to verify separation of upstream development and evaluation patients. The development-wide ICC filter described in S2 was the explicitly identified shared preprocessing step.

**S4 Performance Evaluation and Statistical Implementation**

**Prediction aggregation and discrimination**

Each development patient received five outer out-of-fold probabilities per model. These were averaged to form the primary evaluation dataset. The resulting 690 prediction records per model were not treated as 690 independent patients.

ROC-AUC assigned half credit to tied positive–negative predictions. DeLong covariance estimates were used to construct normal-approximation 95% confidence intervals for the AUC, truncated to [0, 1]. Average precision was calculated as the recall-increment-weighted average of precision values across thresholds. The Brier score was the mean squared difference between predicted probabilities and binary outcomes.

The prespecified primary comparison was M4 versus M2, with the AUC difference defined as M4 minus M2. Secondary comparisons were M2 versus M1, M3 versus M1, and M4 versus M3, with Holm adjustment across their P values. GWO-XGBoost versus Grid-XGBoost was evaluated separately, with the difference defined as GWO minus Grid. Paired AUC differences and confidence intervals were calculated using the joint DeLong covariance rather than by comparing the overlap of separate model confidence intervals.

**Paired bootstrap**

For the difference in Brier score between M4 and M2, 2,000 bootstrap samples were drawn with replacement within the symptomatic and asymptomatic strata while preserving their respective sample sizes. The same sampled patients were used for both models. The 2.5th and 97.5th percentiles defined the 95% confidence interval. The bootstrap seed was 42.

Predictions were fixed: feature selection, tuning, and fitting were not repeated. These intervals, and the DeLong comparisons, did not fully propagate uncertainty from rerunning the entire model-development process.

**Calibration**

Probabilities were clipped to [10^−6^, 1 − 10^−6^] before logit transformation. Calibration-in-the-large was estimated by fitting an intercept while fixing the prediction-logit coefficient at 1. Calibration slope was estimated using logistic regression with a freely estimated intercept and prediction-logit coefficient.

The calibration-in-the-large intercept was therefore distinct from the free intercept in the slope model. Calibration curves used the fitted intercept-and-slope relationship for evaluation without replacing original predictions in other performance calculations. Non-estimable or unstable fits were flagged.

**Classification thresholds**

For each model, finite thresholds were evaluated using patient-averaged development out-of-fold predictions. The threshold maximizing sensitivity + specificity − 1 was selected. Ties favored higher sensitivity, followed by the lower threshold. Predictions at or above the threshold were classified as symptomatic.

The threshold was applied unchanged to the independent internal validation cohort. Sensitivity, specificity, accuracy, PPV, and NPV were calculated from confusion-matrix counts with Wilson 95% confidence intervals. Development-cohort classification measures calculated using a threshold selected from the same predictions were considered descriptive rather than an independent validation of that threshold.

**Decision curve analysis**

Net benefit was calculated at thresholds from 0.05 to 0.80 in steps of 0.01:

$$NB = \frac{\mathrm{TP}}{N} - \frac{\mathrm{FP}}{N} \times\frac{p_{t}}{1 - p_{t}}.$$

Treat-none had a net benefit of zero; treat-all used the observed prevalence and threshold odds. Curves were generated separately using the patient-averaged out-of-fold predictions from the development cohort and the predictions from the independent internal validation cohort. DCA was exploratory and was not used to tune models, select thresholds, or establish the effectiveness of a treatment policy.

**Model interpretation and stability**

Interventional TreeSHAP was applied to the final M3 using all 138 development patients as the background dataset. The raw XGBoost output was explained, corresponding to log-odds under the binary logistic objective. Global importance was ranked by mean absolute SHAP values. Summary, dependence, and waterfall plots described contributions, and additivity was checked against raw model outputs.

Feature-selection frequencies were calculated across 25 outer training partitions. Pairwise Jaccard similarity was the size of the intersection divided by the size of the union of selected feature sets. Repeat-specific AUCs and patient-level prediction variability were also summarized. These repeated estimates shared patients and were not independent replications.

Spearman correlations between original.shape.Maximum2DDiameterSlice and conventional lesion length and width were descriptive supplementary analyses. They were not used as evidence of incremental predictive value.

**Baseline statistics and software**

In the descriptive implementation, continuous variables with absolute sample skewness ≤ 1 were summarized as mean and standard deviation; otherwise, medians and interquartile ranges were used. Continuous group comparisons used two-sided Mann–Whitney *U* tests. Categorical comparisons used Pearson’s chi-square test without continuity correction or Fisher’s exact test when an expected cell count was below five.

Standardized mean differences used the difference in means divided by the square root of the average within-cohort variances; binary variables used the corresponding average Bernoulli variance. Baseline comparisons did not determine predictor inclusion.

The final environment comprised Python 3.13.5, NumPy 2.3.5, pandas 2.3.3, SciPy 1.16.3, scikit-learn 1.8.0, XGBoost 3.1.2, and SHAP 0.50.0. Image processing used the separate software specified in S1.
