# Supplementary material for: Development and internal validation of CTA-based radiomics models for differentiating symptomatic from asymptomatic unruptured spontaneous intracranial vertebrobasilar artery dissection
Source: Front Neurol. 2026 Oct 23;17:1883223. doi: 10.3389/fneur.2026.1883223 (PMC13739547; doi:10.3389/fneur.2026.1883223)
Supplement: Supplementary file 3 [file Data_Sheet_3.ZIP › Supplementary tables/Table_S1_Cohort_balance.docx]

**Supplementary Table S1. Baseline characteristics of the development and independent internal validation cohorts**

| **Variable** | **Development (n = 138)** | **Internal validation (n = 35)** | **SMD** | **Absolute SMD** |
| --- | --- | --- | --- | --- |
| Age | 56.85 ± 9.85 | 55.69 ± 11.07 | 0.111 | 0.111 |
| Sex | 34 (24.6%) | 8 (22.9%) | 0.042 | 0.042 |
| Smoking | 28 (20.3%) | 8 (22.9%) | -0.062 | 0.062 |
| Drinking | 14 (10.1%) | 4 (11.4%) | -0.041 | 0.041 |
| Hypertension | 77 (55.8%) | 22 (62.9%) | -0.144 | 0.144 |
| Diabetes | 21 (15.2%) | 4 (11.4%) | 0.112 | 0.112 |
| Coronary heart disease | 11 (8.0%) | 5 (14.3%) | -0.202 | 0.202 |
| Hyperlipidemia | 15 (10.9%) | 3 (8.6%) | 0.078 | 0.078 |
| Family history | 1 (0.7%) | 0 (0.0%) | 0.121 | 0.121 |
| Cerebral infarction | 28 (20.3%) | 7 (20.0%) | 0.007 | 0.007 |
| SAH | 1 (0.7%) | 1 (2.9%) | -0.161 | 0.161 |
| mRS | 0.00 [0.00, 1.00] | 0.00 [0.00, 0.50] | -0.208 | 0.208 |
| Location | 51 (37.0%) | 13 (37.1%) | -0.004 | 0.004 |
| Length | 9.61 [6.53, 13.47] | 10.00 [6.66, 14.95] | -0.201 | 0.201 |
| Width | 4.92 [3.41, 7.42] | 5.05 [3.83, 8.28] | -0.192 | 0.192 |
| Parent artery stenosis | 10 (7.2%) | 3 (8.6%) | -0.049 | 0.049 |
| Endovascular treatment | 101 (73.2%) | 23 (65.7%) | 0.163 | 0.163 |

Note. SMD denotes standardized mean difference, calculated as development minus internal validation. SMDs describe partition balance and were not used for predictor selection.
