# Supplementary material for: Development and internal validation of CTA-based radiomics models for differentiating symptomatic from asymptomatic unruptured spontaneous intracranial vertebrobasilar artery dissection
Source: Front Neurol. 2026 Oct 23;17:1883223. doi: 10.3389/fneur.2026.1883223 (PMC13739547; doi:10.3389/fneur.2026.1883223)
Supplement: Supplementary file 3 [file Data_Sheet_3.ZIP › Supplementary tables/Table_S2_Final_radiomic_features.docx]

**Supplementary Table S2. Radiomic features retained in the final full-development model**

| **Feature** | **Feature class** | **ICC(2,1) (95% CI)** | **Standardized LASSO coefficient** |
| --- | --- | --- | --- |
| original.shape.Maximum2DDiameterSlice | SHAPE | 0.960 (0.947–0.970) | 0.076579 |
| wavelet-HLH.glszm.SmallAreaEmphasis | GLSZM | 0.911 (0.884–0.933) | 0.009988 |
| wavelet-LLL.gldm.LargeDependenceLowGrayLevelEmphasis | GLDM | 0.834 (0.787–0.873) | 0.148485 |
| wavelet-HHH.firstorder.Skewness | FIRSTORDER | 0.937 (0.918–0.953) | -0.357334 |
| wavelet-HHH.glszm.ZoneVariance | GLSZM | 0.854 (0.813–0.889) | 0.150135 |
| wavelet-LHH.glszm.SizeZoneNonUniformityNormalized | GLSZM | 0.891 (0.859–0.918) | 0.067066 |
| wavelet-HHL.glszm.ZoneVariance | GLSZM | 0.934 (0.914–0.950) | 0.327716 |
| wavelet-HHH.glszm.LowGrayLevelZoneEmphasis | GLSZM | 0.833 (0.786–0.872) | -0.044409 |
| wavelet-HLH.glszm.SizeZoneNonUniformityNormalized | GLSZM | 0.852 (0.809–0.887) | 0.017810 |
| original.firstorder.TotalEnergy | FIRSTORDER | 0.955 (0.940–0.966) | 0.036417 |
| wavelet-HLH.glszm.LowGrayLevelZoneEmphasis | GLSZM | 0.883 (0.849–0.912) | -0.144807 |
| wavelet-HHH.firstorder.Minimum | FIRSTORDER | 0.845 (0.801–0.882) | -0.070468 |
| wavelet-LHL.ngtdm.Busyness | NGTDM | 0.891 (0.859–0.918) | 0.024626 |
| wavelet-HLL.ngtdm.Contrast | NGTDM | 0.928 (0.906–0.946) | 0.019234 |
| wavelet-LHH.glszm.SmallAreaEmphasis | GLSZM | 0.895 (0.864–0.921) | 0.037089 |
| wavelet-LLH.firstorder.MeanAbsoluteDeviation | FIRSTORDER | 0.865 (0.826–0.898) | -0.527803 |
| wavelet-LLH.glrlm.ShortRunLowGrayLevelEmphasis | GLRLM | 0.893 (0.861–0.919) | -0.145254 |
| wavelet-LLH.firstorder.TotalEnergy | FIRSTORDER | 0.946 (0.929–0.959) | 0.147107 |
| wavelet-LLL.firstorder.Energy | FIRSTORDER | 0.845 (0.800–0.882) | 0.076882 |
| wavelet-LHL.gldm.LowGrayLevelEmphasis | GLDM | 0.945 (0.928–0.959) | -0.016185 |

Note. These 20 features were retained when the radiomics pipeline was refitted on all 138 development patients. Outcome-informed selection was repeated independently within each outer-training partition; this list was not imposed on all outer folds.
