# Supplementary material for: Development and internal validation of CTA-based radiomics models for differentiating symptomatic from asymptomatic unruptured spontaneous intracranial vertebrobasilar artery dissection
Source: Front Neurol. 2026 Oct 23;17:1883223. doi: 10.3389/fneur.2026.1883223 (PMC13739547; doi:10.3389/fneur.2026.1883223)
Supplement: Supplementary file 3 [file Data_Sheet_3.ZIP › Supplementary tables/Table_S3_Final_model_parameters.docx]

**Supplementary Table S3. Final model parameters and M4 coefficients**

**A. Selected model parameters**

| **Model** | **Estimator** | **Parameter** | **Selected value** | **Criterion/context** |
| --- | --- | --- | --- | --- |
| M1 | Ridge logistic regression | C | 0.046415888 | Mean inner log-loss 0.609552 |
| M2 | Ridge logistic regression | C | 0.067341507 | Mean inner log-loss 0.603686 |
| M4 | Ridge logistic regression | C | 0.038535286 | Mean inner log-loss 0.596505 |
| M3 | GWO-XGBoost | learning_rate | 0.05500844 | Final full-development fit |
| M3 | GWO-XGBoost | max_depth | 8 | Final full-development fit |
| M3 | GWO-XGBoost | min_child_weight | 10 | Final full-development fit |
| M3 | GWO-XGBoost | subsample | 0.76100534 | Final full-development fit |
| M3 | GWO-XGBoost | colsample_bytree | 0.56205286 | Final full-development fit |
| M3 | GWO-XGBoost | gamma | 1.354041 | Final full-development fit |
| M3 | GWO-XGBoost | reg_alpha | 0.60080116 | Final full-development fit |
| M3 | GWO-XGBoost | reg_lambda | 1.499322 | Final full-development fit |
| M3 | GWO-XGBoost | n_estimators | 193 | Final full-development fit |

**B. Final M4 coefficients**

| **Predictor** | **Standardized coefficient** | **Original-unit coefficient** | **Imputation** |
| --- | --- | --- | --- |
| Intercept |  | -2.28641073 |  |
| Location | 0.10912417 | 0.10912417 | 0 |
| Length | 0.23282222 | 0.03181541 | 9.605 |
| Width | 0.14957579 | 0.04150182 | 4.92 |
| Parent artery stenosis | -0.06515907 | -0.06515907 | 0 |
| Age | -0.00065187 | -0.00006641 | 57 |
| Sex | -0.13650270 | -0.13650270 | 0 |
| Smoking | -0.00660427 | -0.00660427 | 0 |
| Drinking | 0.08620442 | 0.08620442 | 0 |
| Hypertension | 0.21121460 | 0.21121460 | 1 |
| Diabetes | 0.03072035 | 0.03072035 | 0 |
| Coronary heart disease | 0.04234027 | 0.04234027 | 0 |
| Hyperlipidemia | 0.04461656 | 0.04461656 | 0 |
| Radiomics probability | 0.32385265 | 2.41270857 | 0.278106 |

Note. The original-unit equation yields the probability of symptomatic status. Binary variables retain 0/1 coding; parent artery stenosis is coded 0 for absent and 1 for present.
