# Supplementary material for: Development and internal validation of CTA-based radiomics models for differentiating symptomatic from asymptomatic unruptured spontaneous intracranial vertebrobasilar artery dissection
Source: Front Neurol. 2026 Oct 23;17:1883223. doi: 10.3389/fneur.2026.1883223 (PMC13739547; doi:10.3389/fneur.2026.1883223)
Supplement: Supplementary file 3 [file Data_Sheet_3.ZIP › Supplementary tables/Table_S4_GWO_vs_Grid.docx]

**Supplementary Table S4. Formal comparison of GWO-XGBoost and Grid-XGBoost**

| **Comparison/strategy** | **Patient-averaged OOF AUC** | **Candidate evaluations per outer fold** | **Optimization/comparison details** |
| --- | --- | --- | --- |
| GWO-XGBoost | 0.6755 | 1500 | 25 wolves × 60 iterations |
| Grid-XGBoost | 0.6504 | 8748 | Cartesian grid |
| GWO minus Grid | 0.0251 |  | 95% CI -0.0221 to 0.0723; paired DeLong P = 0.2971 |

Note. The strategies used identical outer and inner partitions and the same cached training-specific feature selections. Search budgets differed; the comparison does not establish superiority under equal computational resources.
