# Supplementary material for: Development and internal validation of CTA-based radiomics models for differentiating symptomatic from asymptomatic unruptured spontaneous intracranial vertebrobasilar artery dissection
Source: Front Neurol. 2026 Oct 23;17:1883223. doi: 10.3389/fneur.2026.1883223 (PMC13739547; doi:10.3389/fneur.2026.1883223)
Supplement: Supplementary file 3 [file Data_Sheet_3.ZIP › Supplementary tables/Table_S5_Calibration_and_classification.docx]

**Supplementary Table S5. Calibration and complete locked-threshold classification results**

**A. Calibration metrics**

| **Cohort** | **Model** | **Calibration-in-the-large** | **Calibration slope** | **Free intercept** |
| --- | --- | --- | --- | --- |
| Development OOF | M1 | -0.0068 | 0.8883 | -0.0853 |
| Development OOF | M2 | 0.0183 | 0.9701 | -0.0030 |
| Development OOF | M3 | 0.0606 | 0.9751 | 0.0424 |
| Development OOF | M4 | 0.0138 | 1.1887 | 0.1422 |
| Internal validation | M1 | -0.1517 | 3.3172 | 1.2857 |
| Internal validation | M2 | -0.1886 | 3.4322 | 1.2736 |
| Internal validation | M3 | -0.0033 | 1.3258 | 0.2011 |
| Internal validation | M4 | -0.1638 | 6.4466 | 2.3833 |

**B. Independent internal validation classification metrics**

| **Model** | **Cutoff** | **Metric** | **Estimate (95% CI), %** | **TP** | **FP** | **TN** | **FN** |
| --- | --- | --- | --- | --- | --- | --- | --- |
| M1 | 0.3421 | Sensitivity | 63.636 (35.380–84.834) | 7 | 5 | 19 | 4 |
| M1 | 0.3421 | Specificity | 79.167 (59.530–90.755) | 7 | 5 | 19 | 4 |
| M1 | 0.3421 | Accuracy | 74.286 (57.931–85.837) | 7 | 5 | 19 | 4 |
| M1 | 0.3421 | PPV | 58.333 (31.951–80.674) | 7 | 5 | 19 | 4 |
| M1 | 0.3421 | NPV | 82.609 (62.862–93.021) | 7 | 5 | 19 | 4 |
| M2 | 0.3420 | Sensitivity | 72.727 (43.435–90.254) | 8 | 5 | 19 | 3 |
| M2 | 0.3420 | Specificity | 79.167 (59.530–90.755) | 8 | 5 | 19 | 3 |
| M2 | 0.3420 | Accuracy | 77.143 (60.983–87.934) | 8 | 5 | 19 | 3 |
| M2 | 0.3420 | PPV | 61.538 (35.523–82.290) | 8 | 5 | 19 | 3 |
| M2 | 0.3420 | NPV | 86.364 (66.665–95.251) | 8 | 5 | 19 | 3 |
| M3 | 0.3528 | Sensitivity | 72.727 (43.435–90.254) | 8 | 4 | 20 | 3 |
| M3 | 0.3528 | Specificity | 83.333 (64.147–93.321) | 8 | 4 | 20 | 3 |
| M3 | 0.3528 | Accuracy | 80.000 (64.108–89.958) | 8 | 4 | 20 | 3 |
| M3 | 0.3528 | PPV | 66.667 (39.062–86.188) | 8 | 4 | 20 | 3 |
| M3 | 0.3528 | NPV | 86.957 (67.873–95.462) | 8 | 4 | 20 | 3 |
| M4 | 0.3167 | Sensitivity | 100.000 (74.117–100.000) | 11 | 5 | 19 | 0 |
| M4 | 0.3167 | Specificity | 79.167 (59.530–90.755) | 11 | 5 | 19 | 0 |
| M4 | 0.3167 | Accuracy | 85.714 (70.624–93.740) | 11 | 5 | 19 | 0 |
| M4 | 0.3167 | PPV | 68.750 (44.404–85.835) | 11 | 5 | 19 | 0 |
| M4 | 0.3167 | NPV | 100.000 (83.182–100.000) | 11 | 5 | 19 | 0 |

Note. Calibration-in-the-large was estimated with the prediction-logit coefficient fixed at 1. Classification cutoffs were locked from development OOF predictions. Confidence intervals for classification metrics are Wilson intervals.
