# Supplementary material for: Development and internal validation of CTA-based radiomics models for differentiating symptomatic from asymptomatic unruptured spontaneous intracranial vertebrobasilar artery dissection
Source: Front Neurol. 2026 Oct 23;17:1883223. doi: 10.3389/fneur.2026.1883223 (PMC13739547; doi:10.3389/fneur.2026.1883223)
Supplement: Supplementary file 3 [file Data_Sheet_3.ZIP › Supplementary tables/Table_S6_Repeated_CV_variability.docx]

**Supplementary Table S6. Performance variability across repeated nested cross-validation**

**A. Distribution of AUC across 25 outer folds**

| **Model** | **Mean** | **SD** | **Median** | **Q1** | **Q3** | **Minimum** | **Maximum** |
| --- | --- | --- | --- | --- | --- | --- | --- |
| M1 | 0.6500 | 0.0914 | 0.6173 | 0.5731 | 0.7485 | 0.5309 | 0.7963 |
| M2 | 0.6777 | 0.0754 | 0.6667 | 0.6140 | 0.7251 | 0.5497 | 0.8596 |
| M3 | 0.6646 | 0.0997 | 0.6543 | 0.6080 | 0.7469 | 0.5000 | 0.8304 |
| M4 | 0.7154 | 0.0618 | 0.7193 | 0.6790 | 0.7593 | 0.5731 | 0.8333 |

**B. Patient-level OOF AUC in each repeat**

| **Repeat** | **M1** | **M2** | **M3** | **M4** |
| --- | --- | --- | --- | --- |
| 1 | 0.6330 | 0.6662 | 0.6569 | 0.6996 |
| 2 | 0.6406 | 0.6729 | 0.6870 | 0.7080 |
| 3 | 0.6217 | 0.6896 | 0.6986 | 0.7266 |
| 4 | 0.6191 | 0.6514 | 0.5790 | 0.6834 |
| 5 | 0.6315 | 0.6614 | 0.6349 | 0.6836 |

**C. Within-patient prediction variability across five repeats**

| **Model** | **Group** | **Median SD** | **Q1** | **Q3** | **Mean SD** | **SD of SD** |
| --- | --- | --- | --- | --- | --- | --- |
| M1 | overall | 0.0181 | 0.0113 | 0.0268 | 0.0273 | 0.0280 |
| M1 | group0 | 0.0161 | 0.0110 | 0.0252 | 0.0254 | 0.0264 |
| M1 | group1 | 0.0194 | 0.0138 | 0.0307 | 0.0313 | 0.0309 |
| M2 | overall | 0.0357 | 0.0263 | 0.0514 | 0.0403 | 0.0211 |
| M2 | group0 | 0.0359 | 0.0261 | 0.0501 | 0.0405 | 0.0219 |
| M2 | group1 | 0.0342 | 0.0269 | 0.0520 | 0.0399 | 0.0194 |
| M3 | overall | 0.0969 | 0.0717 | 0.1304 | 0.1047 | 0.0499 |
| M3 | group0 | 0.0857 | 0.0667 | 0.1179 | 0.0984 | 0.0475 |
| M3 | group1 | 0.1025 | 0.0773 | 0.1444 | 0.1177 | 0.0528 |
| M4 | overall | 0.0602 | 0.0410 | 0.0814 | 0.0683 | 0.0382 |
| M4 | group0 | 0.0589 | 0.0416 | 0.0765 | 0.0661 | 0.0358 |
| M4 | group1 | 0.0678 | 0.0385 | 0.0947 | 0.0729 | 0.0430 |

Note. Outer folds and repeats share patients and are not independent replications. Patient prediction variability summarizes the standard deviation of each patient's five OOF probabilities.
