# Supplementary material for: Development and internal validation of CTA-based radiomics models for differentiating symptomatic from asymptomatic unruptured spontaneous intracranial vertebrobasilar artery dissection
Source: Front Neurol. 2026 Oct 23;17:1883223. doi: 10.3389/fneur.2026.1883223 (PMC13739547; doi:10.3389/fneur.2026.1883223)
Supplement: Supplementary file 3 [file Data_Sheet_3.ZIP › Supplementary tables/Table_S7_Feature_stability_and_SHAP.docx]

**Supplementary Table S7. Radiomic feature-selection stability and final-model SHAP importance**

**A. Features selected in at least one outer-training partition**

| **Feature** | **Selected outer partitions** | **Frequency** | **Sign consistency** | **Median LASSO coefficient** |
| --- | --- | --- | --- | --- |
| wavelet-HHH.glszm.ZoneVariance | 17 | 0.68 | 1.00 | 0.151239 |
| wavelet-HHL.glszm.ZoneVariance | 16 | 0.64 | 1.00 | 0.314450 |
| wavelet-HHH.firstorder.Skewness | 15 | 0.60 | 1.00 | -0.280269 |
| wavelet-LHH.glszm.SizeZoneNonUniformityNormalized | 15 | 0.60 | 1.00 | 0.137215 |
| wavelet-LLL.gldm.LargeDependenceLowGrayLevelEmphasis | 13 | 0.52 | 1.00 | 0.171995 |
| wavelet-LLH.firstorder.MeanAbsoluteDeviation | 11 | 0.44 | 1.00 | -0.573391 |
| wavelet-LLH.glrlm.ShortRunLowGrayLevelEmphasis | 9 | 0.36 | 1.00 | -0.073928 |
| wavelet-HHH.glszm.LowGrayLevelZoneEmphasis | 8 | 0.32 | 1.00 | -0.082964 |
| wavelet-HHH.firstorder.Energy | 7 | 0.28 | 1.00 | 0.385864 |
| wavelet-HLH.glszm.SizeZoneNonUniformity | 7 | 0.28 | 1.00 | 0.076085 |
| original.shape.Maximum2DDiameterSlice | 6 | 0.24 | 1.00 | 0.193511 |
| wavelet-HHH.glszm.SmallAreaLowGrayLevelEmphasis | 6 | 0.24 | 1.00 | -0.046048 |
| wavelet-HHL.gldm.GrayLevelNonUniformity | 6 | 0.24 | 1.00 | 0.284049 |
| wavelet-HLH.glszm.SizeZoneNonUniformityNormalized | 6 | 0.24 | 1.00 | 0.110208 |
| wavelet-HLH.glszm.SmallAreaEmphasis | 6 | 0.24 | 1.00 | 0.097604 |
| wavelet-LLL.glrlm.RunVariance | 6 | 0.24 | 1.00 | 0.208232 |
| wavelet-HHH.firstorder.Minimum | 5 | 0.20 | 1.00 | -0.167948 |
| wavelet-HLH.glszm.LowGrayLevelZoneEmphasis | 5 | 0.20 | 1.00 | -0.159908 |
| wavelet-LHL.firstorder.Skewness | 5 | 0.20 | 1.00 | -0.142728 |
| original.firstorder.TotalEnergy | 4 | 0.16 | 1.00 | 0.141290 |
| wavelet-HHH.glrlm.ShortRunLowGrayLevelEmphasis | 4 | 0.16 | 1.00 | -0.064971 |
| wavelet-HHL.glrlm.ShortRunLowGrayLevelEmphasis | 4 | 0.16 | 1.00 | -0.105966 |
| wavelet-LHH.glszm.SmallAreaEmphasis | 4 | 0.16 | 1.00 | 0.175149 |
| wavelet-LHL.gldm.GrayLevelVariance | 4 | 0.16 | 1.00 | -0.283100 |
| wavelet-LHL.ngtdm.Busyness | 4 | 0.16 | 1.00 | 0.022560 |
| wavelet-LLH.firstorder.RobustMeanAbsoluteDeviation | 4 | 0.16 | 1.00 | -0.063877 |
| original.shape.Sphericity | 3 | 0.12 | 1.00 | -0.072131 |
| original.shape.SurfaceArea | 3 | 0.12 | 1.00 | 0.241881 |
| wavelet-HHL.ngtdm.Busyness | 3 | 0.12 | 1.00 | 0.106466 |
| wavelet-HLL.ngtdm.Contrast | 3 | 0.12 | 1.00 | 0.047571 |
| wavelet-LLH.firstorder.InterquartileRange | 3 | 0.12 | 1.00 | -0.059949 |
| wavelet-LLH.firstorder.Variance | 3 | 0.12 | 1.00 | -0.107765 |
| wavelet-LLH.glrlm.LowGrayLevelRunEmphasis | 3 | 0.12 | 1.00 | -0.142862 |
| wavelet-LLL.glszm.LowGrayLevelZoneEmphasis | 3 | 0.12 | 1.00 | -0.532151 |
| wavelet-LLL.ngtdm.Busyness | 3 | 0.12 | 1.00 | 0.099999 |
| wavelet-HHH.glcm.ClusterShade | 2 | 0.08 | 1.00 | -0.096499 |
| wavelet-HHH.gldm.LowGrayLevelEmphasis | 2 | 0.08 | 1.00 | -0.062869 |
| wavelet-HHL.glszm.GrayLevelNonUniformity | 2 | 0.08 | 1.00 | 0.056495 |
| wavelet-HHL.glszm.SmallAreaEmphasis | 2 | 0.08 | 1.00 | 0.066615 |
| wavelet-LHL.gldm.LowGrayLevelEmphasis | 2 | 0.08 | 1.00 | -0.112638 |
| wavelet-LLH.firstorder.TotalEnergy | 2 | 0.08 | 1.00 | 0.162494 |
| wavelet-LLH.glcm.Contrast | 2 | 0.08 | 1.00 | -0.116719 |
| wavelet-LLL.firstorder.Energy | 2 | 0.08 | 1.00 | 0.133979 |
| wavelet-LLL.firstorder.RootMeanSquared | 2 | 0.08 | 1.00 | -0.115987 |
| original.firstorder.Energy | 1 | 0.04 | 1.00 | 0.142591 |
| original.firstorder.Kurtosis | 1 | 0.04 | 1.00 | 0.009603 |
| original.firstorder.Maximum | 1 | 0.04 | 1.00 | 0.111047 |
| original.glcm.ClusterProminence | 1 | 0.04 | 1.00 | 0.596566 |
| original.gldm.DependenceNonUniformity | 1 | 0.04 | 1.00 | 0.107850 |
| original.gldm.HighGrayLevelEmphasis | 1 | 0.04 | 1.00 | -0.097456 |
| original.gldm.LargeDependenceLowGrayLevelEmphasis | 1 | 0.04 | 1.00 | 0.040594 |
| original.gldm.SmallDependenceLowGrayLevelEmphasis | 1 | 0.04 | 1.00 | -0.216575 |
| original.glrlm.RunLengthNonUniformity | 1 | 0.04 | 1.00 | 0.047879 |
| original.glszm.GrayLevelNonUniformity | 1 | 0.04 | 1.00 | 0.034707 |
| original.shape.Maximum3DDiameter | 1 | 0.04 | 1.00 | 0.059096 |
| original.shape.MinorAxisLength | 1 | 0.04 | 1.00 | 0.158049 |
| wavelet-HHH.firstorder.TotalEnergy | 1 | 0.04 | 1.00 | 0.199883 |
| wavelet-HHH.glcm.MCC | 1 | 0.04 | 1.00 | -0.028350 |
| wavelet-HHH.gldm.DependenceVariance | 1 | 0.04 | 1.00 | 0.040117 |
| wavelet-HHH.glrlm.GrayLevelNonUniformity | 1 | 0.04 | 1.00 | 0.076039 |
| wavelet-HHH.glszm.GrayLevelNonUniformity | 1 | 0.04 | 1.00 | 0.191646 |
| wavelet-HHL.firstorder.Mean | 1 | 0.04 | 1.00 | -0.489250 |
| wavelet-HHL.gldm.LowGrayLevelEmphasis | 1 | 0.04 | 1.00 | -0.036423 |
| wavelet-HHL.glszm.SizeZoneNonUniformity | 1 | 0.04 | 1.00 | 0.042643 |
| wavelet-HLH.firstorder.Maximum | 1 | 0.04 | 1.00 | -0.062573 |
| wavelet-HLH.gldm.DependenceVariance | 1 | 0.04 | 1.00 | -0.085572 |
| wavelet-HLH.glszm.ZoneEntropy | 1 | 0.04 | 1.00 | 0.008762 |
| wavelet-HLH.ngtdm.Contrast | 1 | 0.04 | 1.00 | 0.187364 |
| wavelet-HLL.firstorder.RobustMeanAbsoluteDeviation | 1 | 0.04 | 1.00 | 0.023659 |
| wavelet-HLL.gldm.DependenceVariance | 1 | 0.04 | 1.00 | -0.249370 |
| wavelet-HLL.gldm.LargeDependenceHighGrayLevelEmphasis | 1 | 0.04 | 1.00 | -0.069762 |
| wavelet-HLL.gldm.SmallDependenceLowGrayLevelEmphasis | 1 | 0.04 | 1.00 | -0.004171 |
| wavelet-HLL.glrlm.RunLengthNonUniformity | 1 | 0.04 | 1.00 | 0.214028 |
| wavelet-LHL.firstorder.90Percentile | 1 | 0.04 | 1.00 | -0.032066 |
| wavelet-LHL.firstorder.Median | 1 | 0.04 | 1.00 | 0.085453 |
| wavelet-LHL.firstorder.TotalEnergy | 1 | 0.04 | 1.00 | 0.157438 |
| wavelet-LHL.glcm.DifferenceVariance | 1 | 0.04 | 1.00 | -0.885035 |
| wavelet-LHL.glcm.SumSquares | 1 | 0.04 | 1.00 | -0.340594 |
| wavelet-LHL.gldm.HighGrayLevelEmphasis | 1 | 0.04 | 1.00 | -0.086862 |
| wavelet-LHL.glrlm.LongRunLowGrayLevelEmphasis | 1 | 0.04 | 1.00 | -0.015860 |
| wavelet-LHL.glrlm.LowGrayLevelRunEmphasis | 1 | 0.04 | 1.00 | -0.190297 |
| wavelet-LHL.glrlm.ShortRunLowGrayLevelEmphasis | 1 | 0.04 | 1.00 | -0.134578 |
| wavelet-LHL.glszm.LargeAreaHighGrayLevelEmphasis | 1 | 0.04 | 1.00 | 0.234488 |
| wavelet-LHL.glszm.LargeAreaLowGrayLevelEmphasis | 1 | 0.04 | 1.00 | 0.324873 |
| wavelet-LHL.glszm.LowGrayLevelZoneEmphasis | 1 | 0.04 | 1.00 | -0.197624 |
| wavelet-LHL.glszm.SmallAreaLowGrayLevelEmphasis | 1 | 0.04 | 1.00 | -0.089824 |
| wavelet-LLH.glcm.DifferenceVariance | 1 | 0.04 | 1.00 | -0.007387 |
| wavelet-LLH.glcm.Id | 1 | 0.04 | 1.00 | 0.091609 |
| wavelet-LLH.gldm.DependenceVariance | 1 | 0.04 | 1.00 | -0.213044 |
| wavelet-LLH.gldm.LowGrayLevelEmphasis | 1 | 0.04 | 1.00 | -0.194137 |
| wavelet-LLH.glszm.LargeAreaEmphasis | 1 | 0.04 | 1.00 | 0.129621 |
| wavelet-LLH.glszm.SizeZoneNonUniformity | 1 | 0.04 | 1.00 | 0.041718 |
| wavelet-LLL.glcm.Contrast | 1 | 0.04 | 1.00 | -0.071275 |
| wavelet-LLL.glszm.SmallAreaLowGrayLevelEmphasis | 1 | 0.04 | 1.00 | -0.183899 |

**B. SHAP importance in the final full-development M3 model**

| **Feature** | **Mean absolute SHAP value** |
| --- | --- |
| wavelet-HHH.glszm.ZoneVariance | 0.448061 |
| wavelet-LLL.gldm.LargeDependenceLowGrayLevelEmphasis | 0.316355 |
| wavelet-LHH.glszm.SizeZoneNonUniformityNormalized | 0.183188 |
| wavelet-HHL.glszm.ZoneVariance | 0.132486 |
| original.shape.Maximum2DDiameterSlice | 0.131348 |
| wavelet-HLH.glszm.SmallAreaEmphasis | 0.110739 |
| wavelet-HLH.glszm.SizeZoneNonUniformityNormalized | 0.098562 |
| wavelet-HHH.firstorder.Skewness | 0.096581 |
| wavelet-HLL.ngtdm.Contrast | 0.093732 |
| wavelet-HHH.glszm.LowGrayLevelZoneEmphasis | 0.087187 |
| wavelet-HHH.firstorder.Minimum | 0.085054 |
| wavelet-LLH.firstorder.MeanAbsoluteDeviation | 0.060036 |
| wavelet-LLH.glrlm.ShortRunLowGrayLevelEmphasis | 0.026536 |
| wavelet-LLH.firstorder.TotalEnergy | 0.018674 |
| original.firstorder.TotalEnergy | 0.016987 |
| wavelet-HLH.glszm.LowGrayLevelZoneEmphasis | 0.000000 |
| wavelet-LHL.ngtdm.Busyness | 0.000000 |
| wavelet-LHH.glszm.SmallAreaEmphasis | 0.000000 |
| wavelet-LLL.firstorder.Energy | 0.000000 |
| wavelet-LHL.gldm.LowGrayLevelEmphasis | 0.000000 |

**C. Pairwise Jaccard similarity among outer-partition feature sets**

| **Pairs** | **Mean** | **SD** | **Median** | **Q1** | **Q3** | **Minimum** | **Maximum** |
| --- | --- | --- | --- | --- | --- | --- | --- |
| 300 | 0.1493 | 0.0996 | 0.1364 | 0.0825 | 0.2000 | 0.0000 | 0.5333 |

Note. Panel A omits features never selected in any of the 25 outer-training partitions. SHAP values represent predictive contributions on the raw XGBoost log-odds scale and were not used for feature selection.
