# Supplementary material for: Development and internal validation of CTA-based radiomics models for differentiating symptomatic from asymptomatic unruptured spontaneous intracranial vertebrobasilar artery dissection
Source: Front Neurol. 2026 Oct 23;17:1883223. doi: 10.3389/fneur.2026.1883223 (PMC13739547; doi:10.3389/fneur.2026.1883223)
Supplement: Supplementary file 3 [file Data_Sheet_3.ZIP › Supplementary tables/Table_S8_Shape_feature_correlations.docx]

**Supplementary Table S8. Correlation of the retained shape feature with conventional CTA dimensions**

| **Conventional CTA measure** | **Radiomic shape feature** | **n** | **Spearman ρ** | **Exploratory P** |
| --- | --- | --- | --- | --- |
| Length | original.shape.Maximum2DDiameterSlice | 138 | 0.0258 | 0.763 |
| Width | original.shape.Maximum2DDiameterSlice | 138 | 0.0229 | 0.789 |

Note. Two-sided Spearman correlations were exploratory and were not used as evidence of incremental predictive value.
